# Supplementary material for: Altertoxins with Quorum Sensing Inhibitory Activities from The Marine-Derived Fungus Cladosporium sp. KFD33
Source: Mar Drugs. 2020 Jan 19;18(1):67. doi: 10.3390/md18010067 (PMC7024320; doi:10.3390/md18010067)
Supplement: Supplementary file 1 [file marinedrugs-18-00067-s001.pdf]

Supporting Information

Altertoxins with quorum sensing inhibitory activities from the marine-derived fungus *Cladosporium* sp. KFD33

Fei Zhang[1,2,†], Li-Man Zhou[1,†], Fan-Dong Kong[1,†], Qing-Yun Ma[1], Qing-Yi Xie[1], Jiu-Hui Li[1], Hao-Fu Dai[1], Lei Guo[2,*], You-Xing Zhao[1,*]

[1]Hainan Key Laboratory of Research and Development of Natural Product from Li Folk Medicine, Institute of Tropical Bioscience and Biotechnology, Chinese Academy of Tropical Agricultural Sciences, Haikou 571101, China; MarchFay@163.com (F. Z); zhouliman88@126.com (L.M. Z); kongfandong@itbb.org.cn (F.D. K); maqingyun@itbb.org.cn (Q.Y. M); xieqingyi@itbb.org.cn (Q.Y. X); lijiuhui@itbb.org.cn (J.H. L.); daihaofu@itbb.org.cn (H.F. D.);

[2]Jiangsu Key Laboratory of Marine Bioresources and Environment, Co-innovation Center of Jiangsu Marine Bio-industry Technology, Jiangsu Ocean University, Lianyungang 222005;

* Correspondence: zhaoyouxing@itbb.org.cn (Y.X. Z); guol@jou.edu.cn (L. G);
Tel.: +86-898-6698-9095 (Y.X. Z); +86-159-5072-6013 (L. G)

† These authors contributed equally to this paper.

List of Supporting Information

Figure S1. The 1H NMR Spectrum of Compound **1** in DMSO 3
Figure S2. The 13C NMR Spectrum of Compound **1** in DMSO 4
Figure S3. The HMQC Spectrum of Compound **1** in DMSO 5
Figure S4. The HMBC Spectrum of Compound **1** in DMSO 6
Figure S5. The COSY Spectrum of Compound **1** in DMSO 7
Figure S6. The HRESIMS Spectroscopic Data of Compound **1** 8
Figure S7. The IR Spectrum of Compound **1** 9
Figure S8. The 1H NMR Spectrum of Compounds **2** and **3** in DMSO 10
Figure S9. The 13C NMR Spectrum of Compounds **2** and **3** in DMSO 11
Figure S10. The DEPT Spectrum of Compounds **2** and **3** in DMSO 12
Figure S11. The HMQC Spectrum of Compounds **2** and **3** in DMSO 13
Figure S12. The HMBC Spectrum of Compounds **2** and **3** in DMSO 14
Figure S13. The COSY Spectrum of Compounds **2** and **3** in DMSO 15
Figure S14. The HRESIMS Spectroscopic Data of Compounds **2** and **3** 16
Figure S15. The IR Spectrum of Compounds **2** and **3** 17
Figure S16. The 1H NMR Spectrum of Compound **4** in DMSO 18

Figure S17. The 13C NMR Spectrum of Compound **4** in DMSO 19
Figure S18. The DEPT Spectrum of Compound **4** in DMSO..20
Figure S19. The HMQC Spectrum of Compound **4** in DMSO 21
Figure S20. The HMBC Spectrum of Compound **4** in DMSO..22
Figure S21. The COSY Spectrum of Compound **4** in DMSO 23
Figure S22. The HRESIMS Spectroscopic Data of Compound **4** 24
Figure S23. The IR Spectrum of Compound **4** 25
Figure S24. The 1H NMR Spectrum of Compound **5** in DMSO 26
Figure S25. The 13C NMR Spectrum of Compound **5** in DMSO 27
Figure S26. The DEPT Spectrum of Compound **5** in DMSO..28
Figure S27. The HMQC Spectrum of Compound **5** in DMSO 29
Figure S28. The HMBC Spectrum of Compound **5** in DMSO..30
Figure S29. The COSY Spectrum of Compound **5** in DMSO 31
Figure S30. The HRESIMS Spectroscopic Data of Compound **5** 32
Figure S31. The IR Spectrum of Compound **5** 33
Figure S32. A peak area ratio of compounds **2** and **3** over a chiral column 34
Figure S33. The strain of *Cladosporium* sp. KFD33..35
Figure S34. C. *violaceum* CV026 well diffusion assay..36
Figure S35. The energy minimized 3D chemical structures for **1**-**5**. 37
The 18*S* gene sequences of *Cladosporium* sp. KFD33 38
Theory and Calculation Details. 39

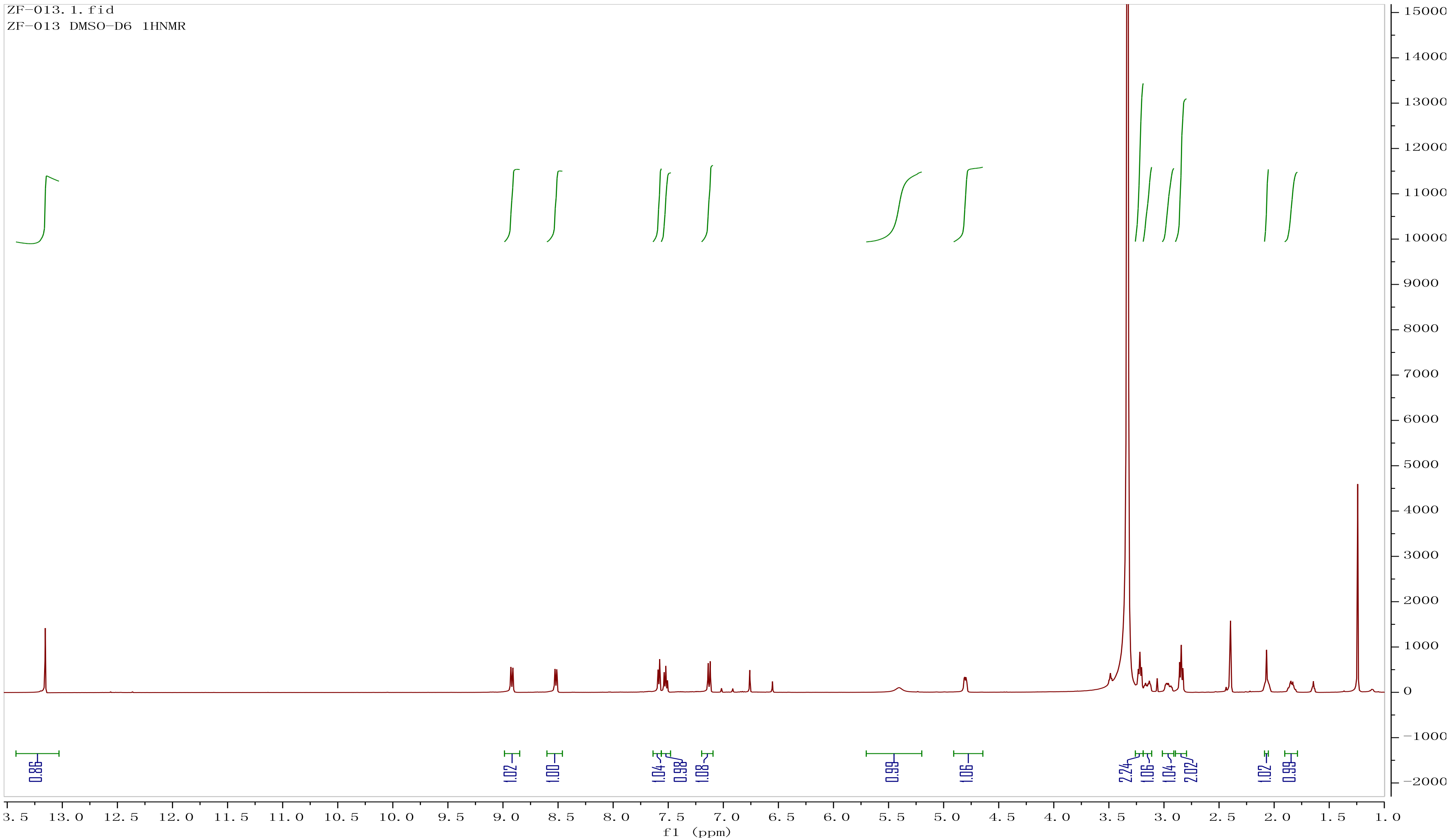

Figure S1. The ¹H NMR Spectrum of Compound **1** in DMSO

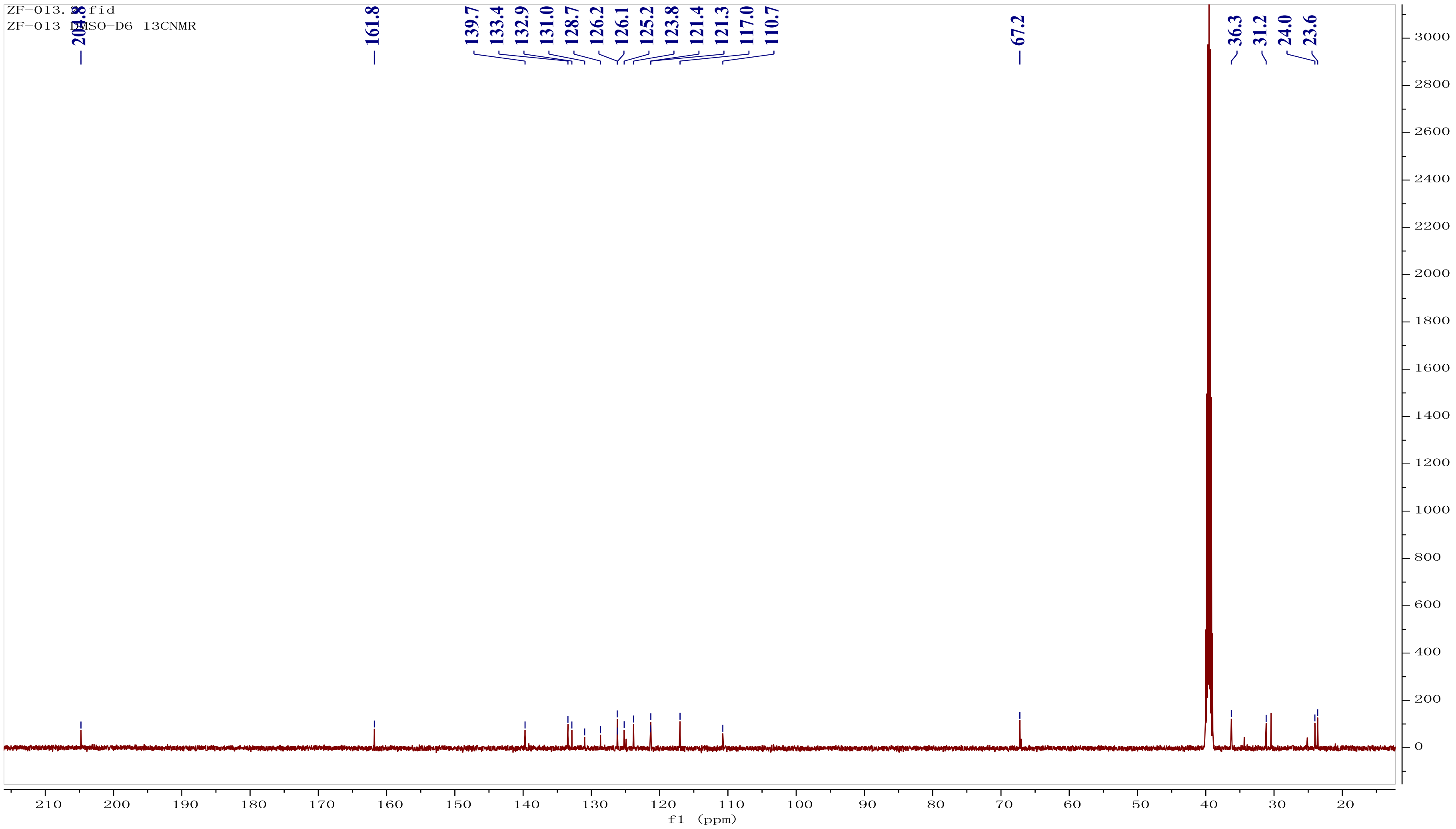

Figure S2. The ^{13}C NMR Spectrum of Compound **1** in DMSO

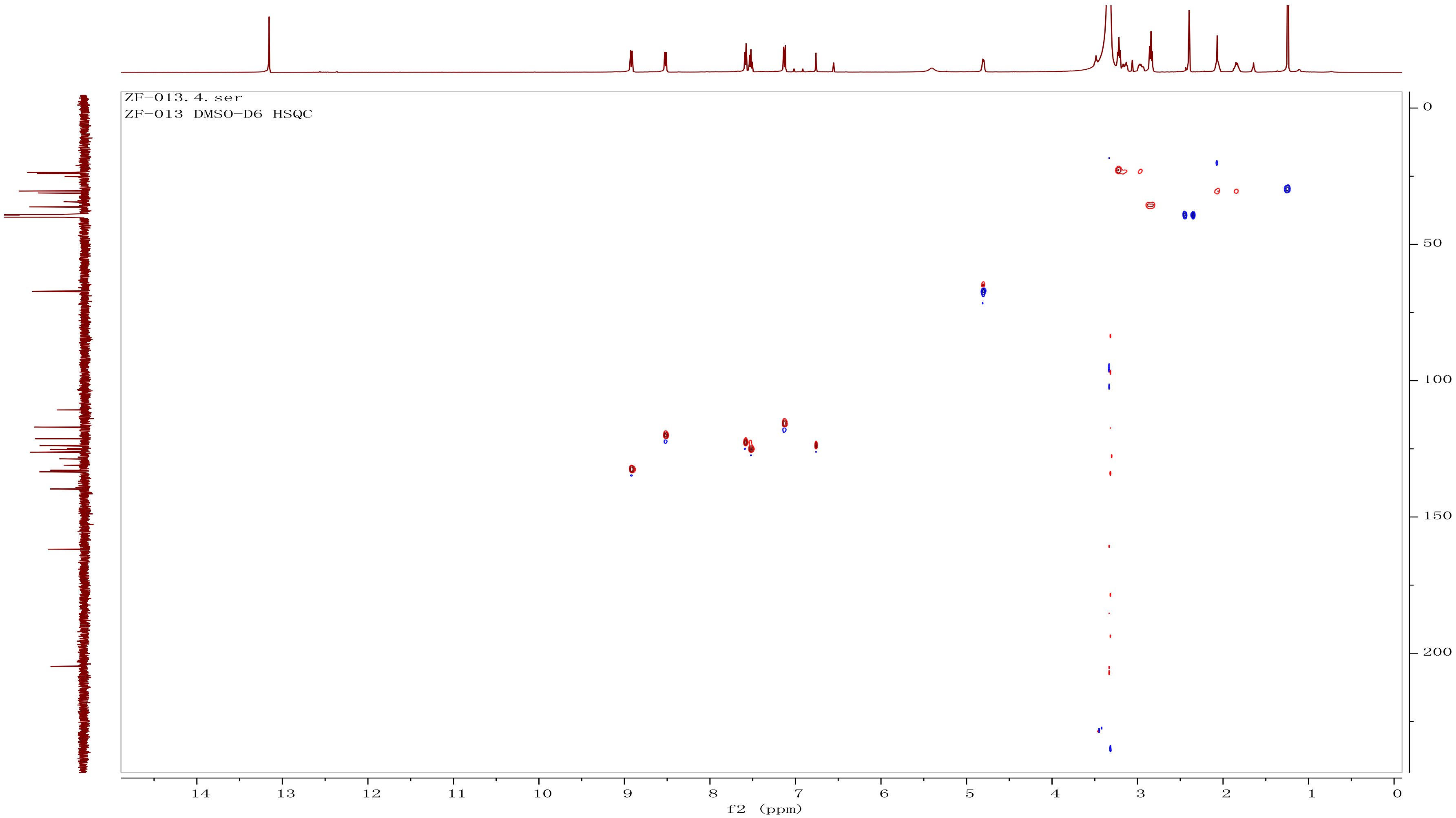

Figure S3. The HMQC Spectrum of Compound **1** in DMSO

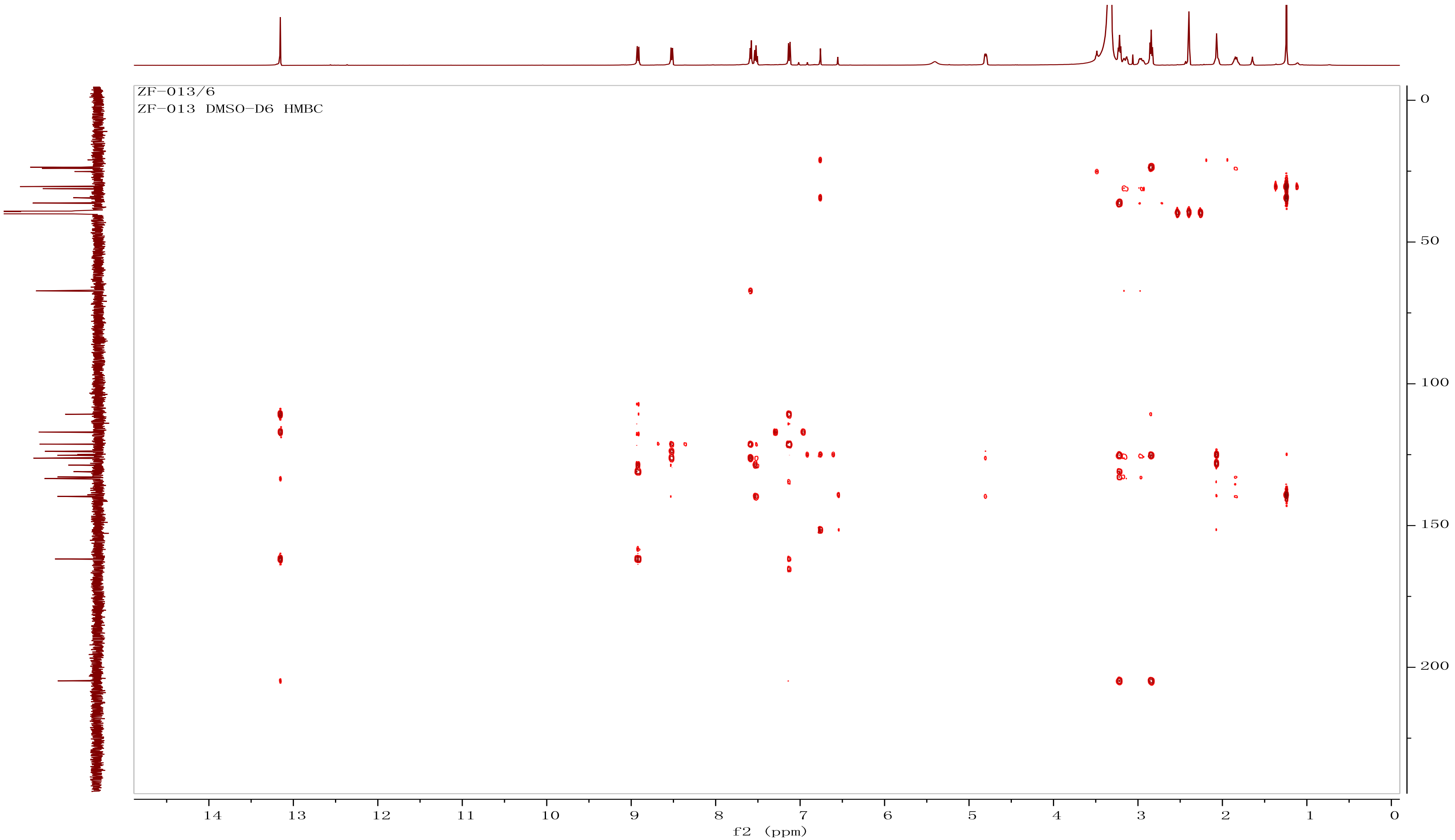

Figure S4. The HMBC Spectrum of Compound **1** in DMSO

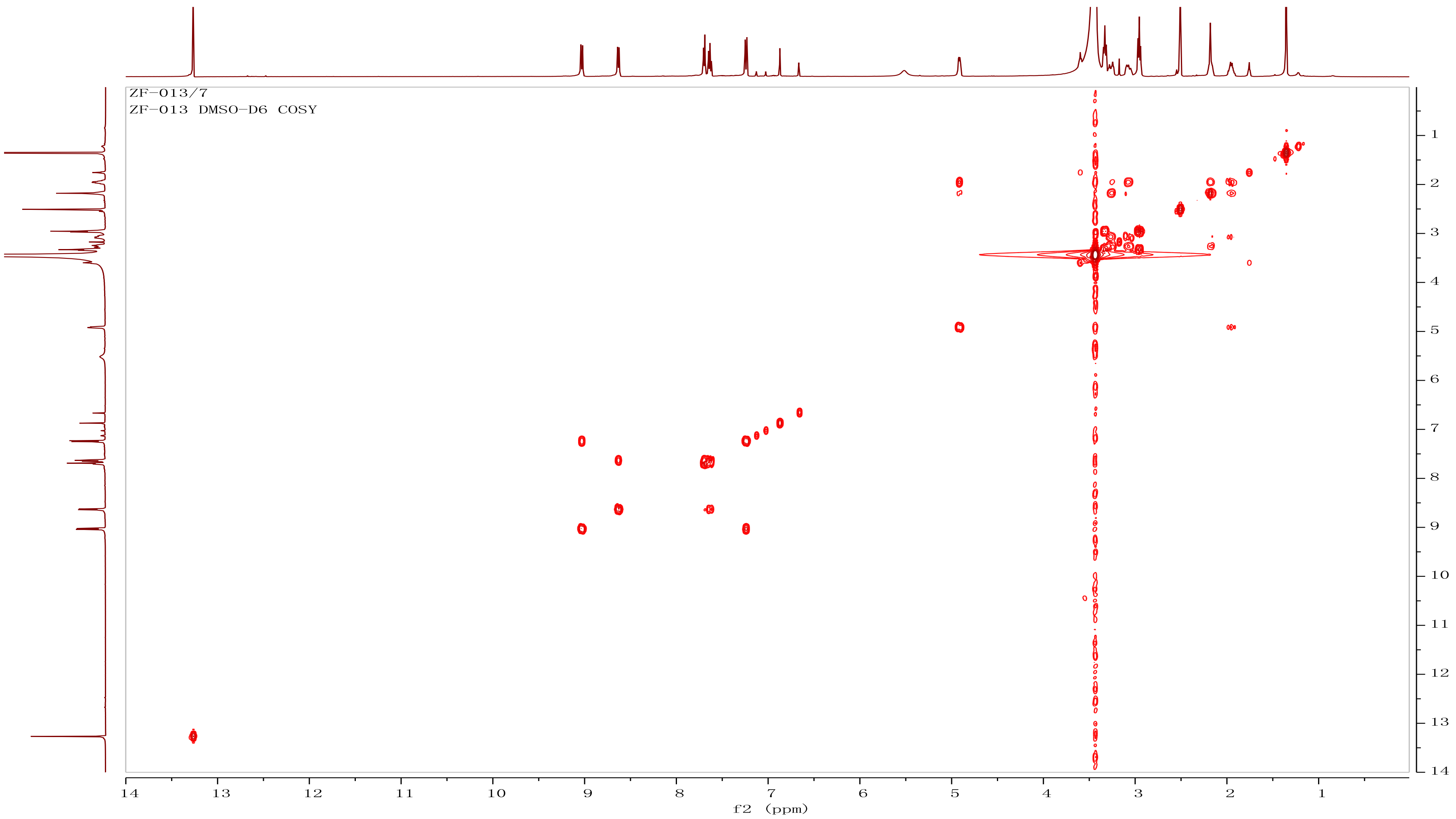

Figure S5. The COSY Spectrum of Compound **1** in DMSO

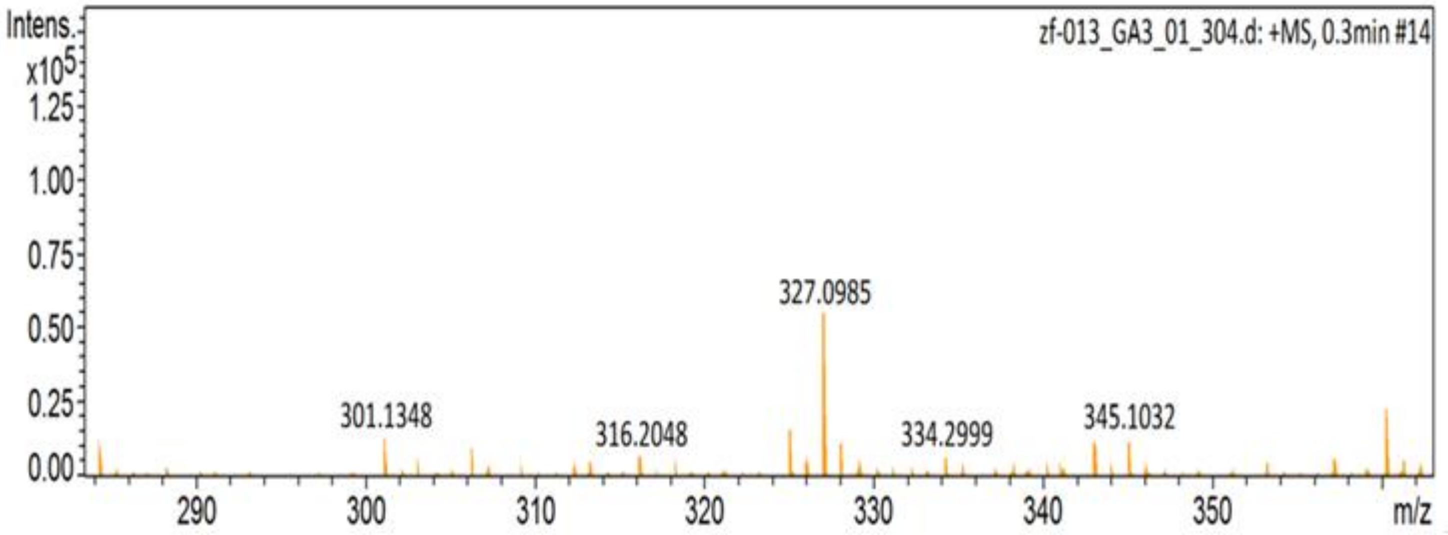

Figure S6. The HRESIMS Spectroscopic Data of Compound **1**

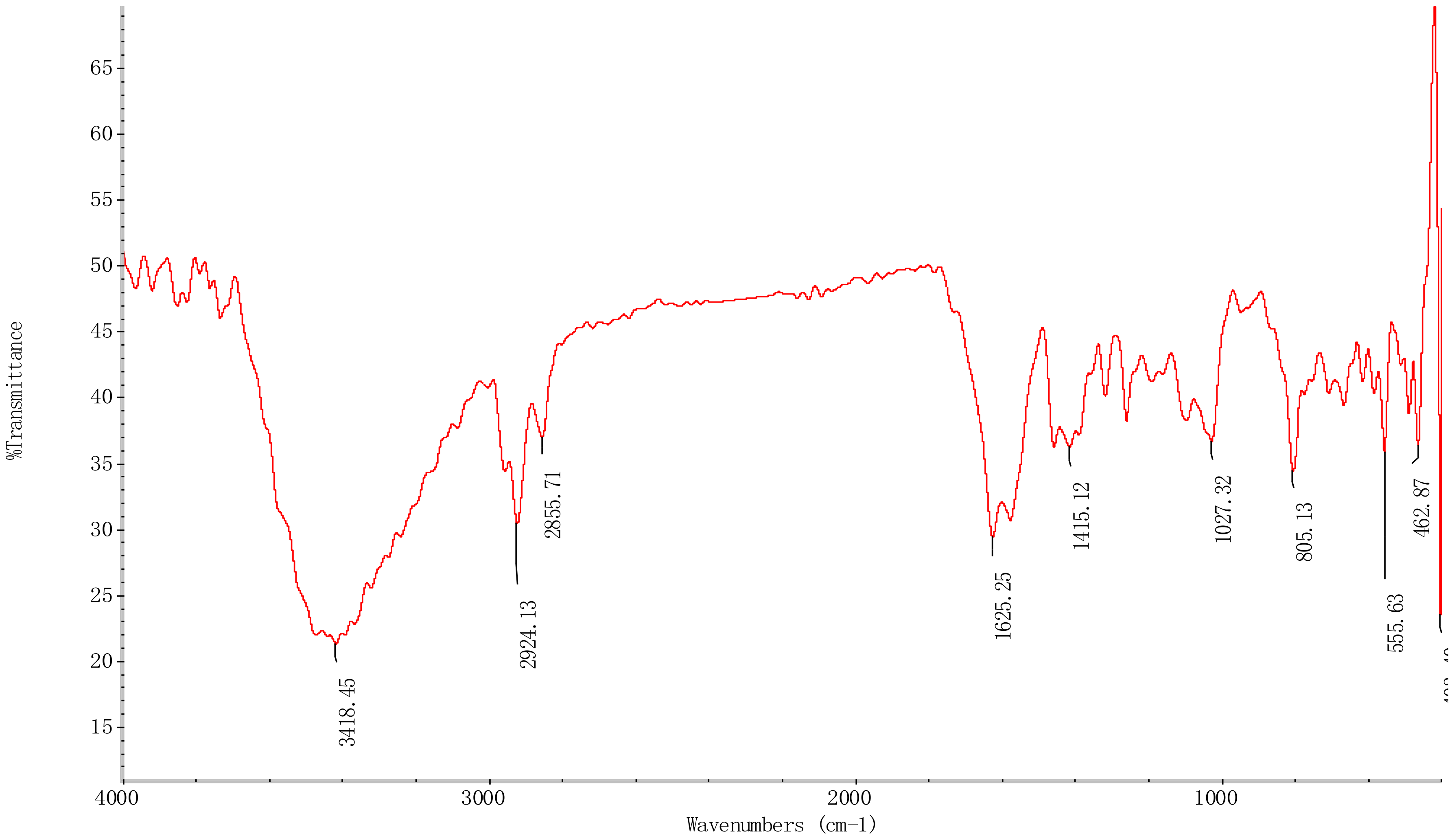

Figure S7. The IR Spectrum of Compound **1**

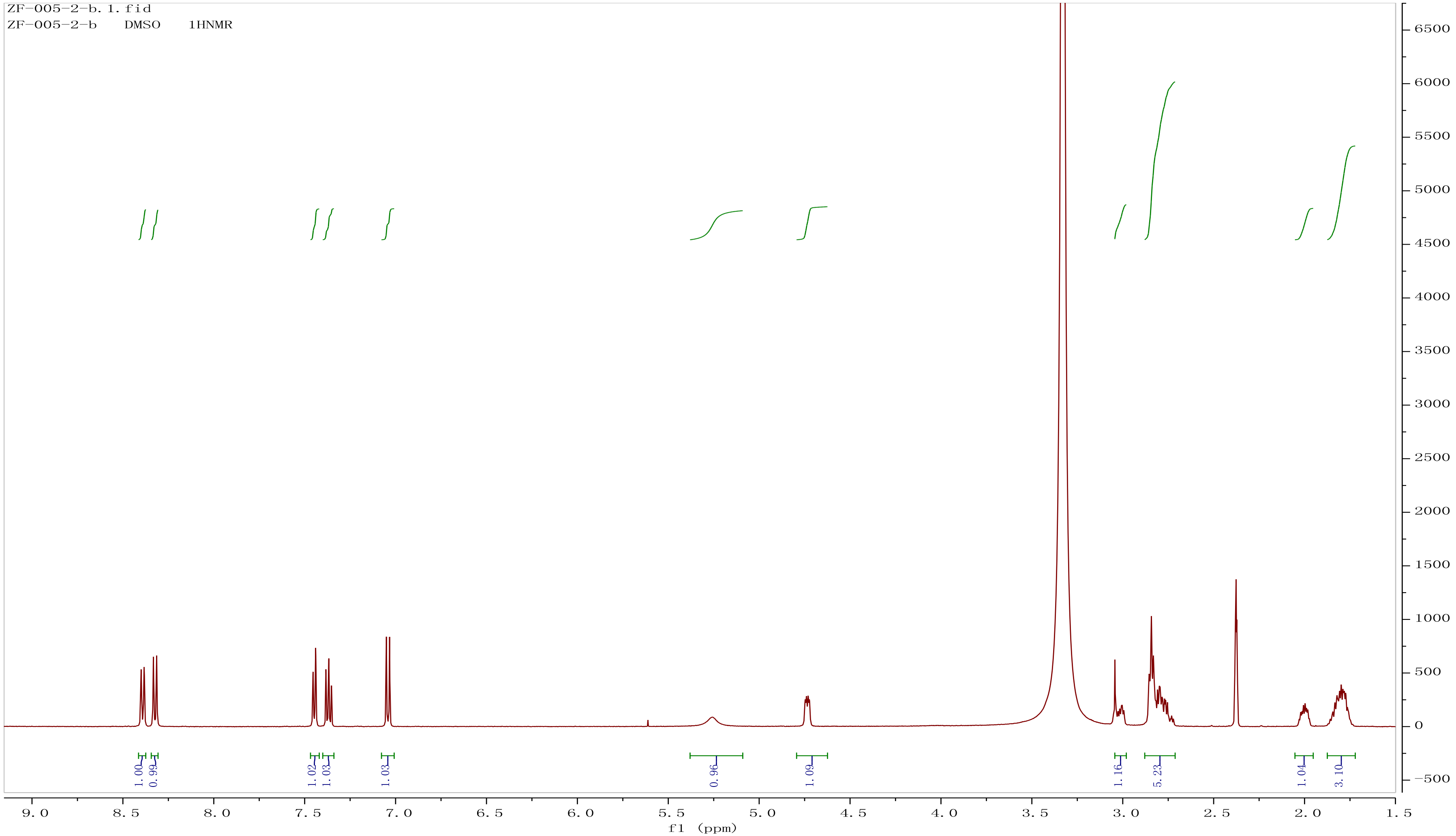

Figure S8. The ^{1}H NMR Spectrum of Compounds **2** and **3** in DMSO

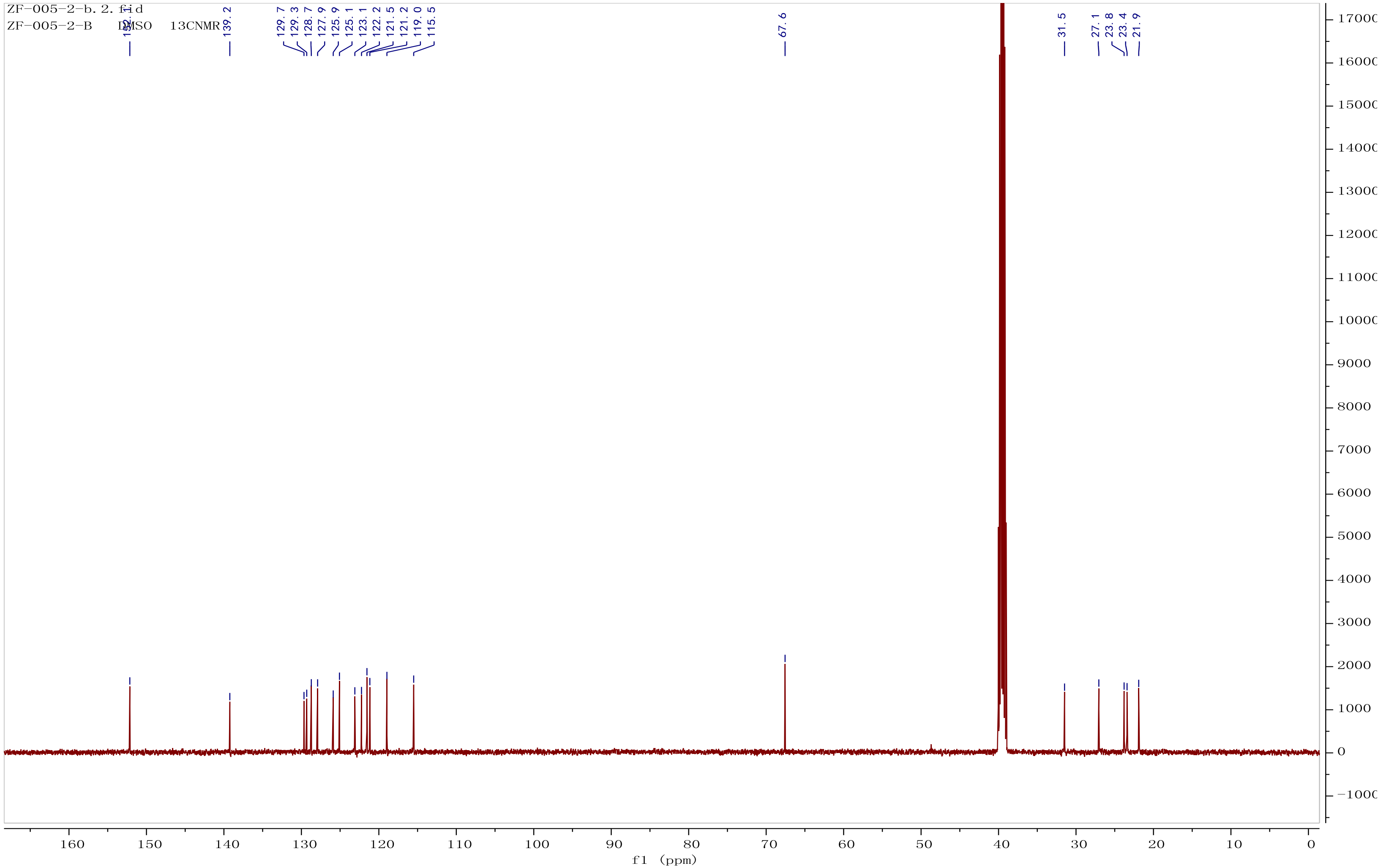

Figure S9. The ^{13}C NMR Spectrum of Compounds **2** and **3** in DMSO

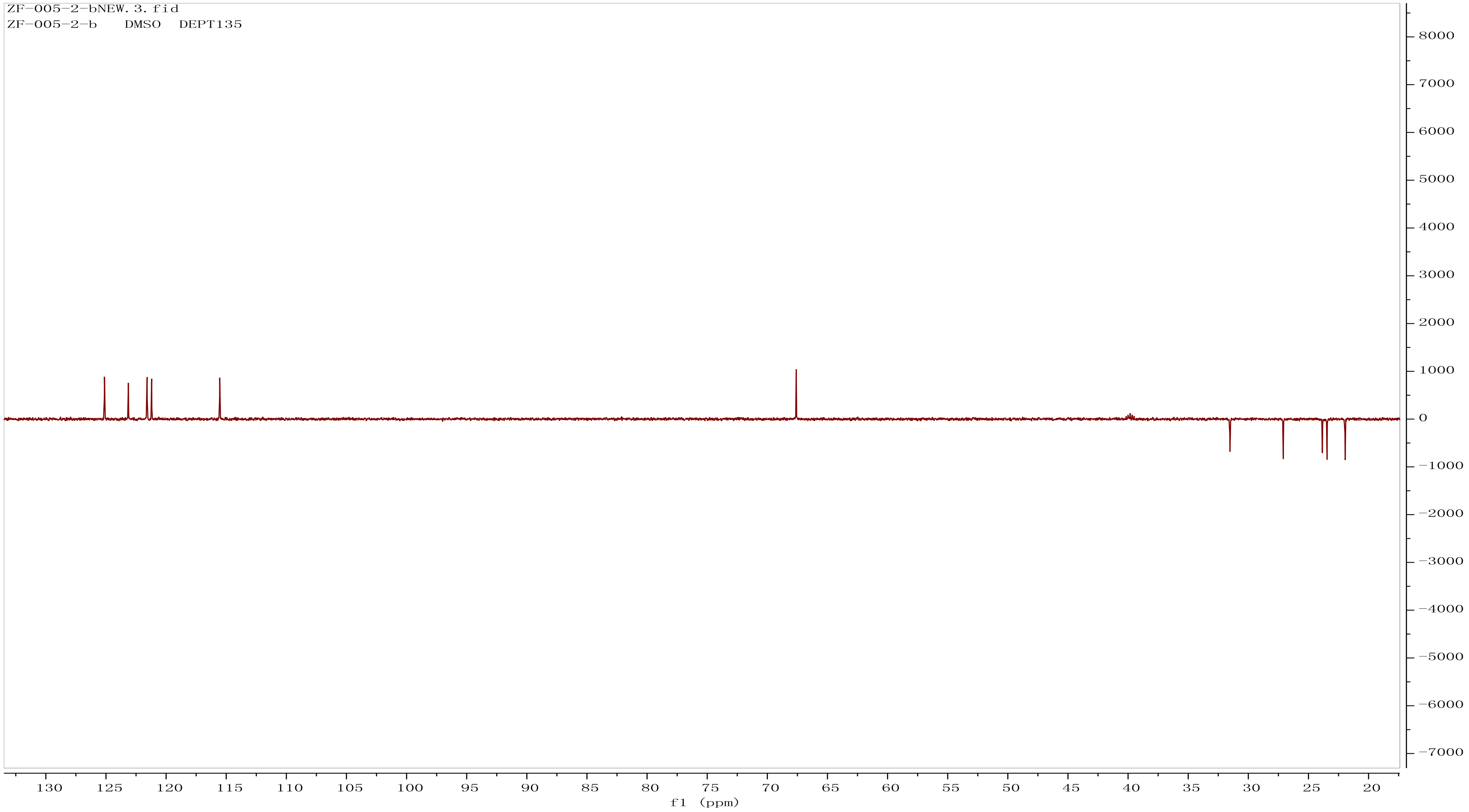

Figure S10. The DEPT Spectrum of Compounds **2** and **3** in DMSO

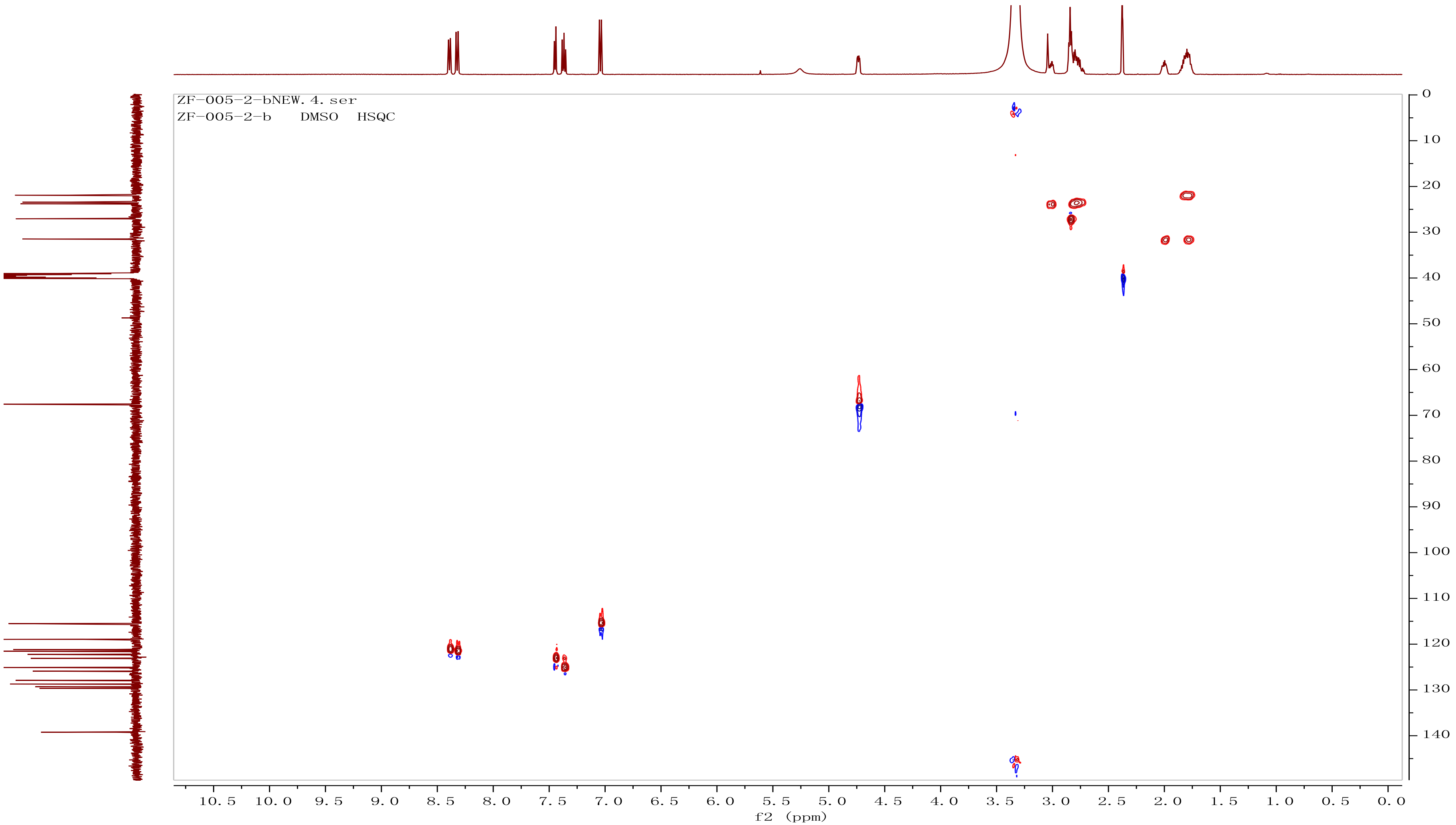

Figure S11. The HMQC Spectrum of Compounds **2** and **3** in DMSO

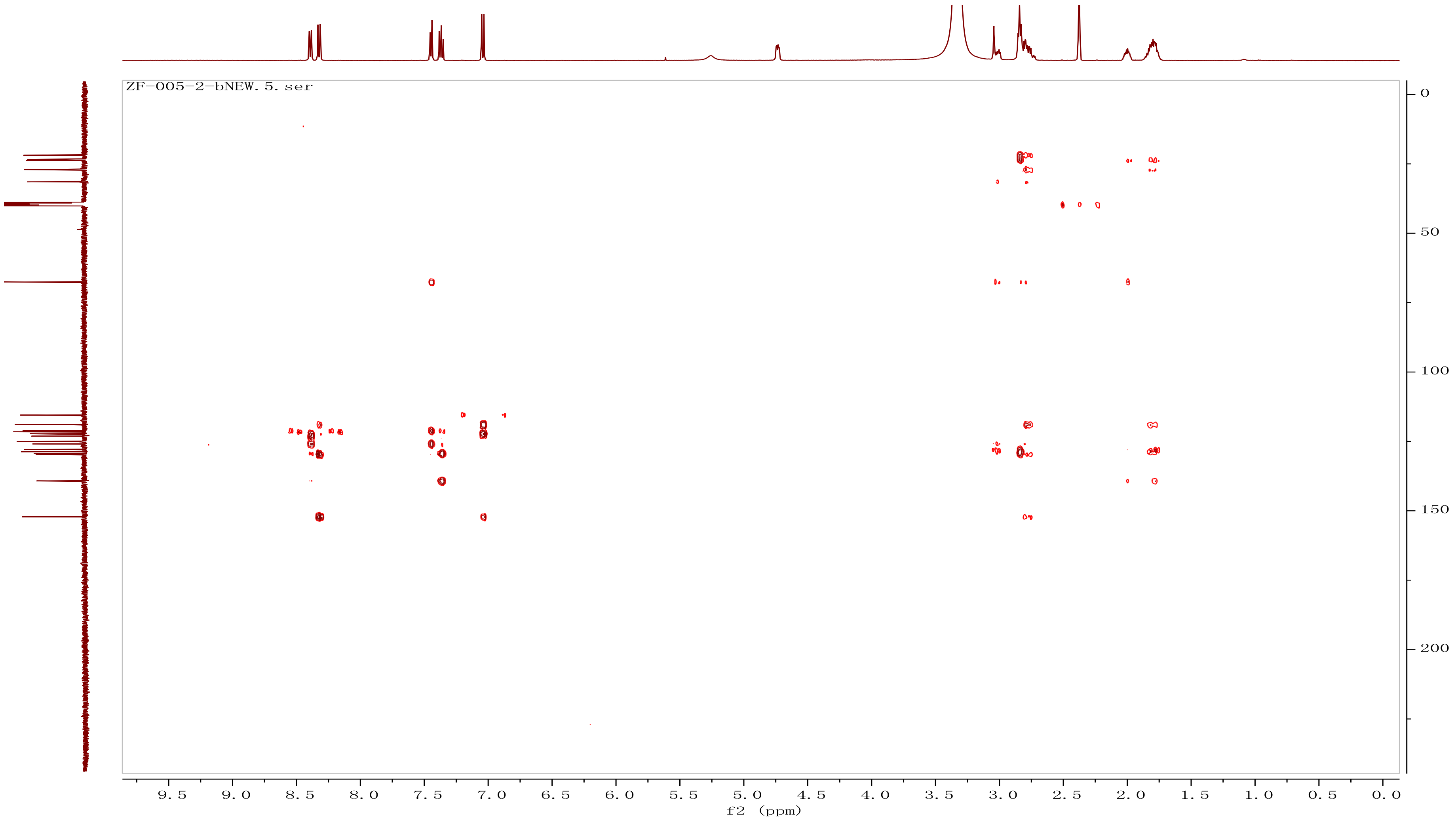

Figure S12. The HMBC Spectrum of Compounds **2** and **3** in DMSO

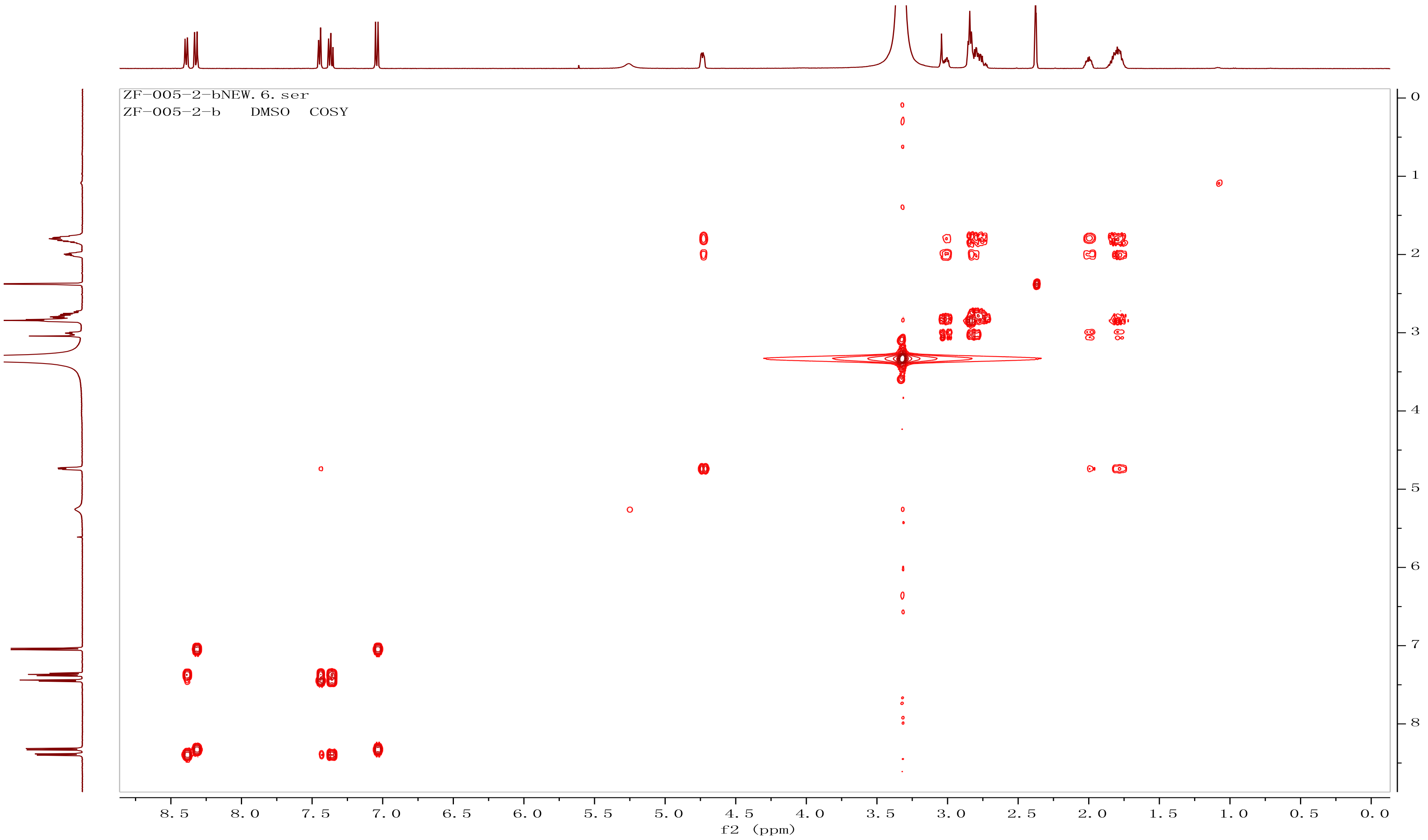

Figure S13. The COSY Spectrum of Compounds **2** and **3** in DMSO

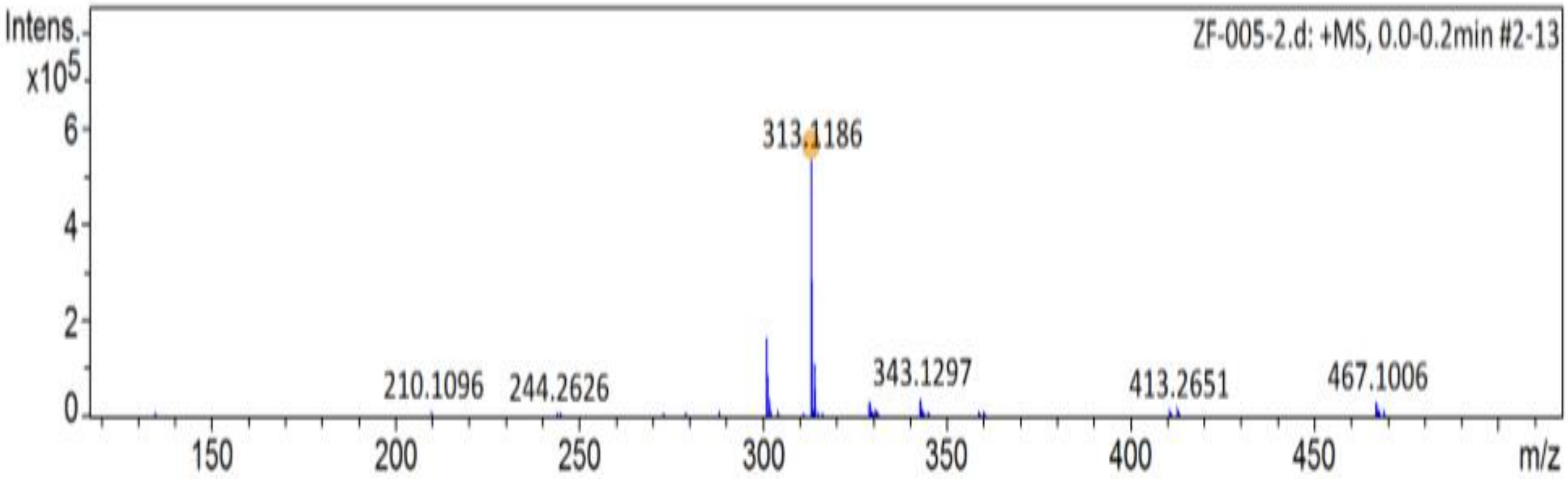

Figure S14. The HRESIMS Spectroscopic Data of Compounds **2** and **3**

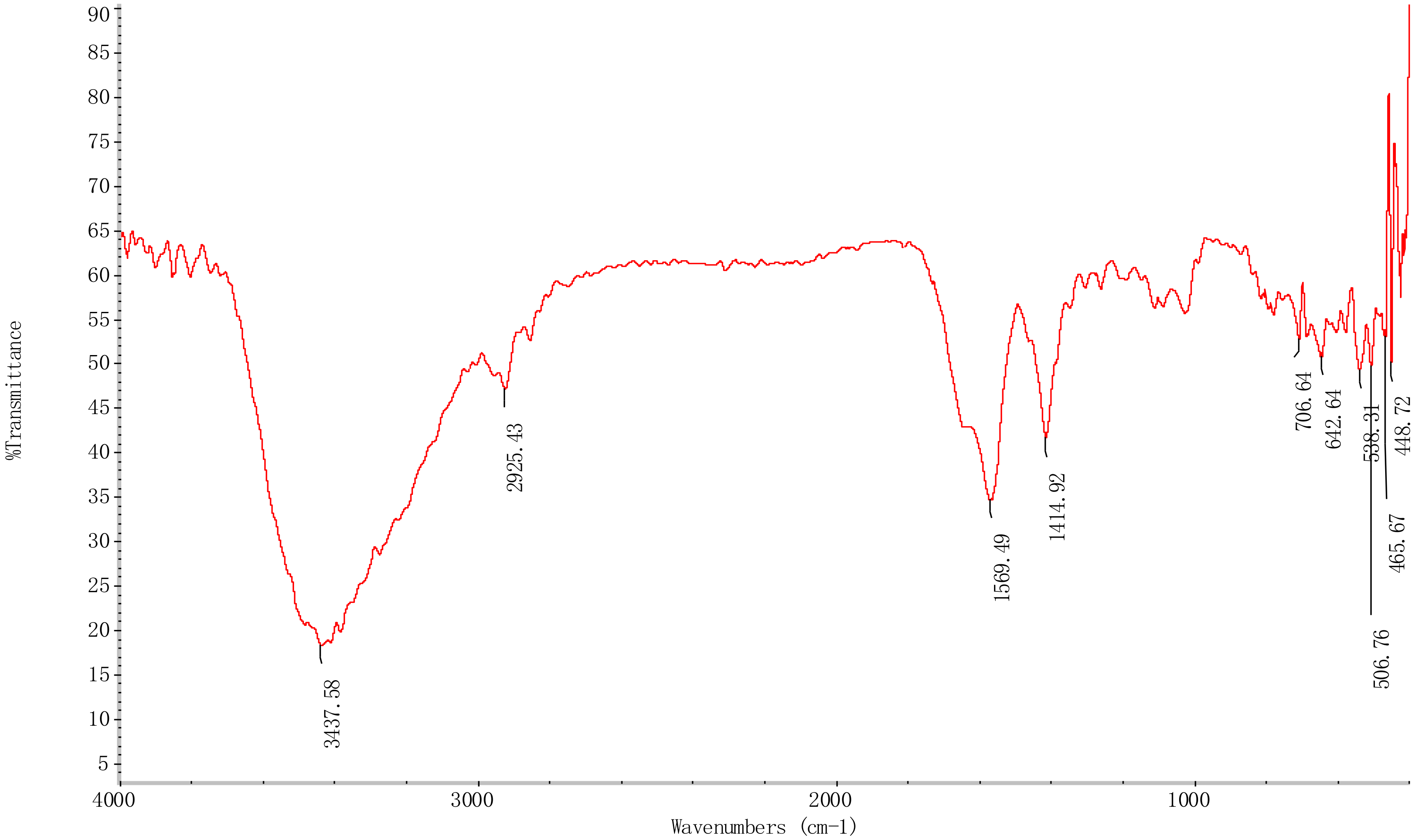

Figure S15. The IR Spectrum of Compounds **2** and **3**

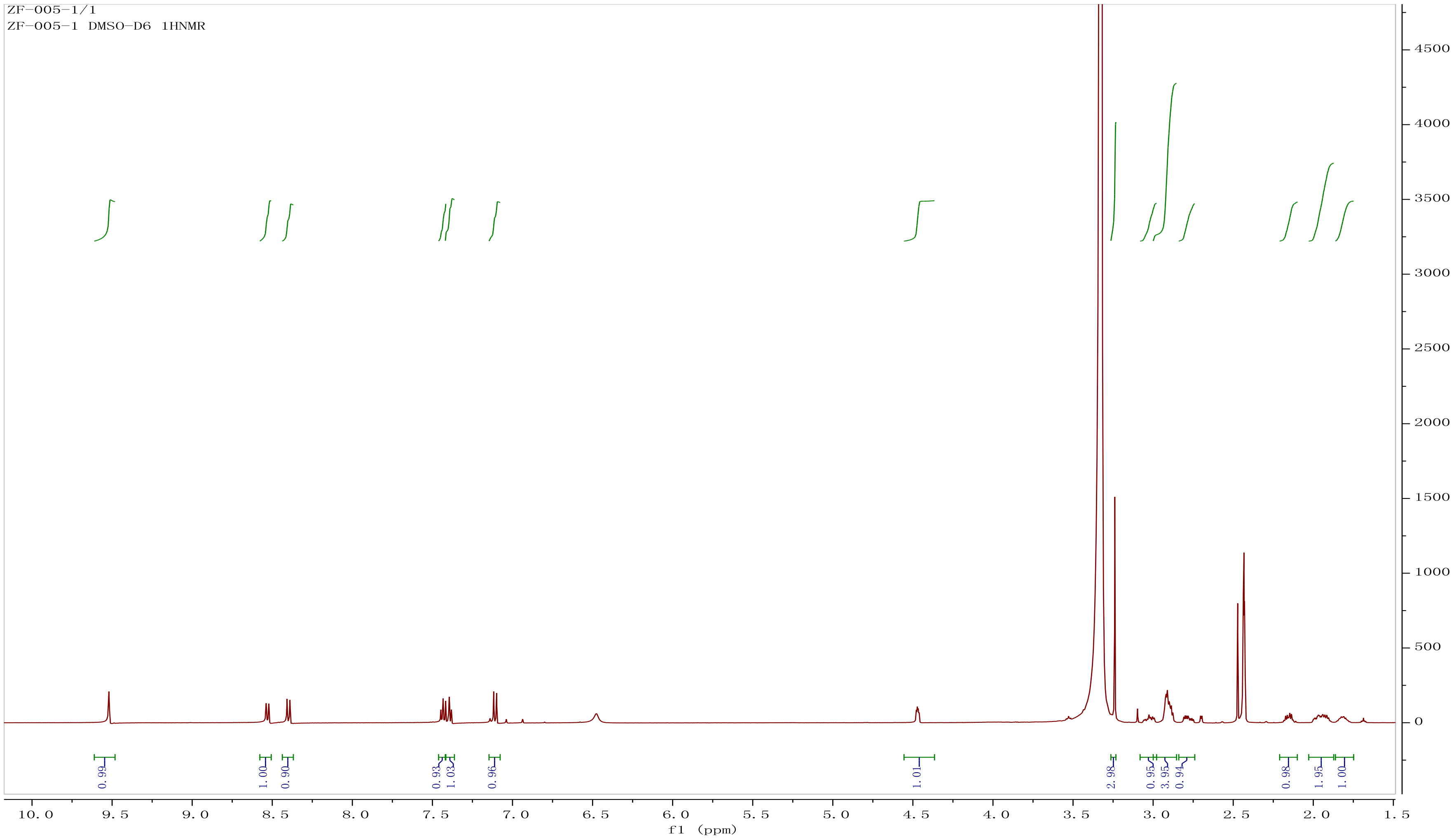

Figure S16. The ^{1}H NMR Spectrum of Compound **4** in DMSO

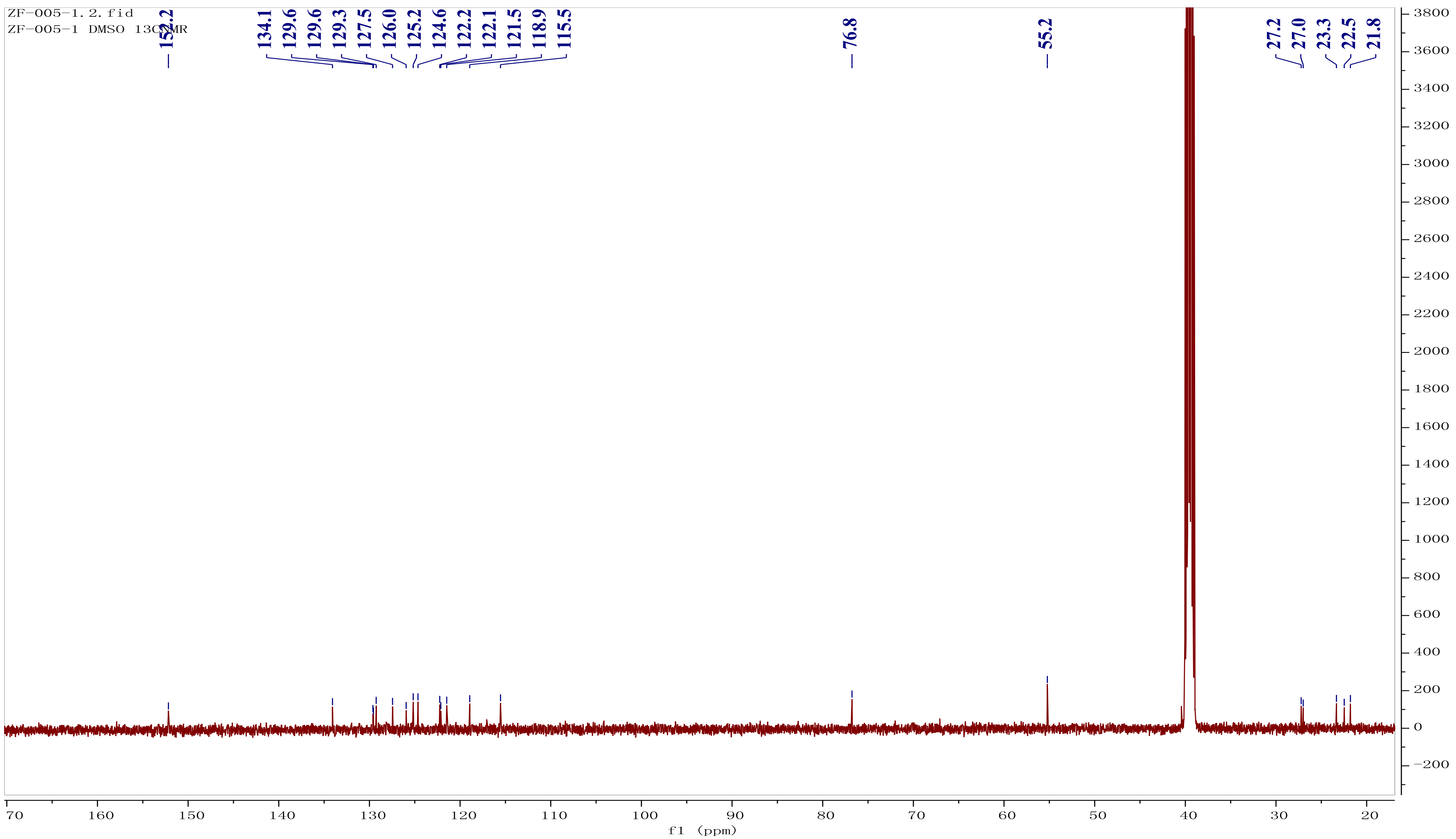

Figure S17. The ^{13}C NMR Spectrum of Compound **4** in DMSO

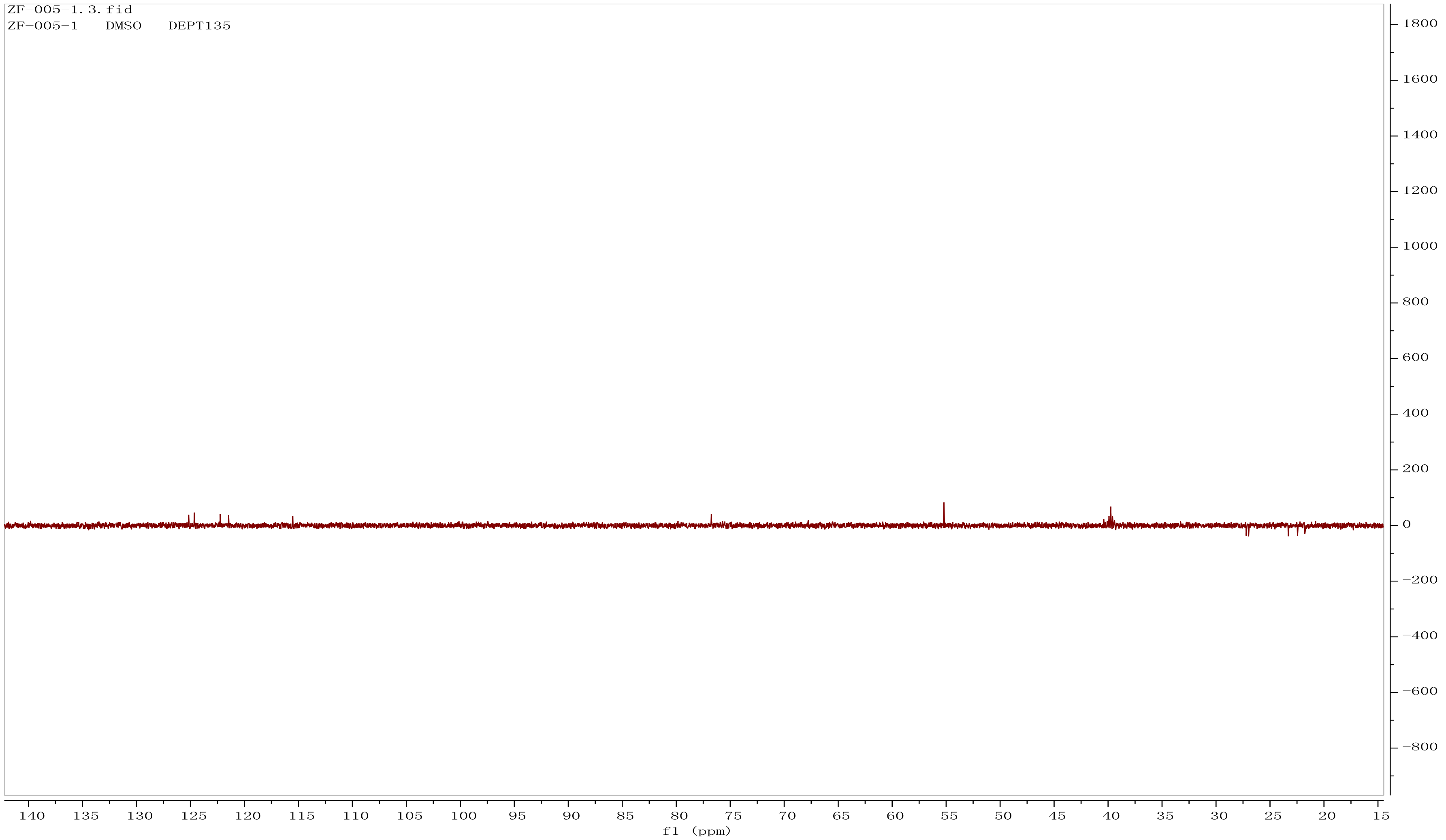

Figure S18. The DEPT Spectrum of Compound **4** in DMSO

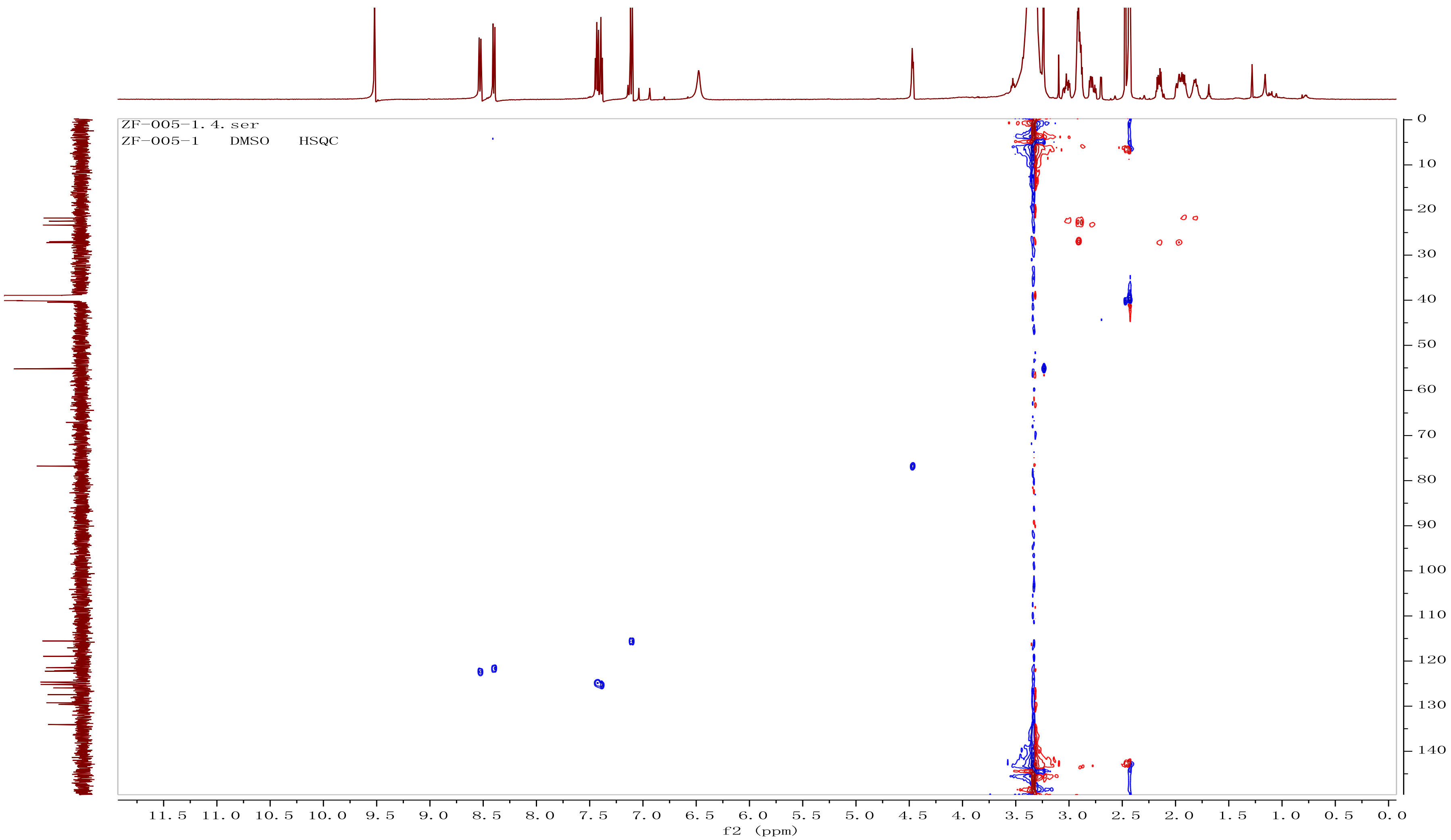

Figure S19. The HMQC Spectrum of Compound **4** in DMSO

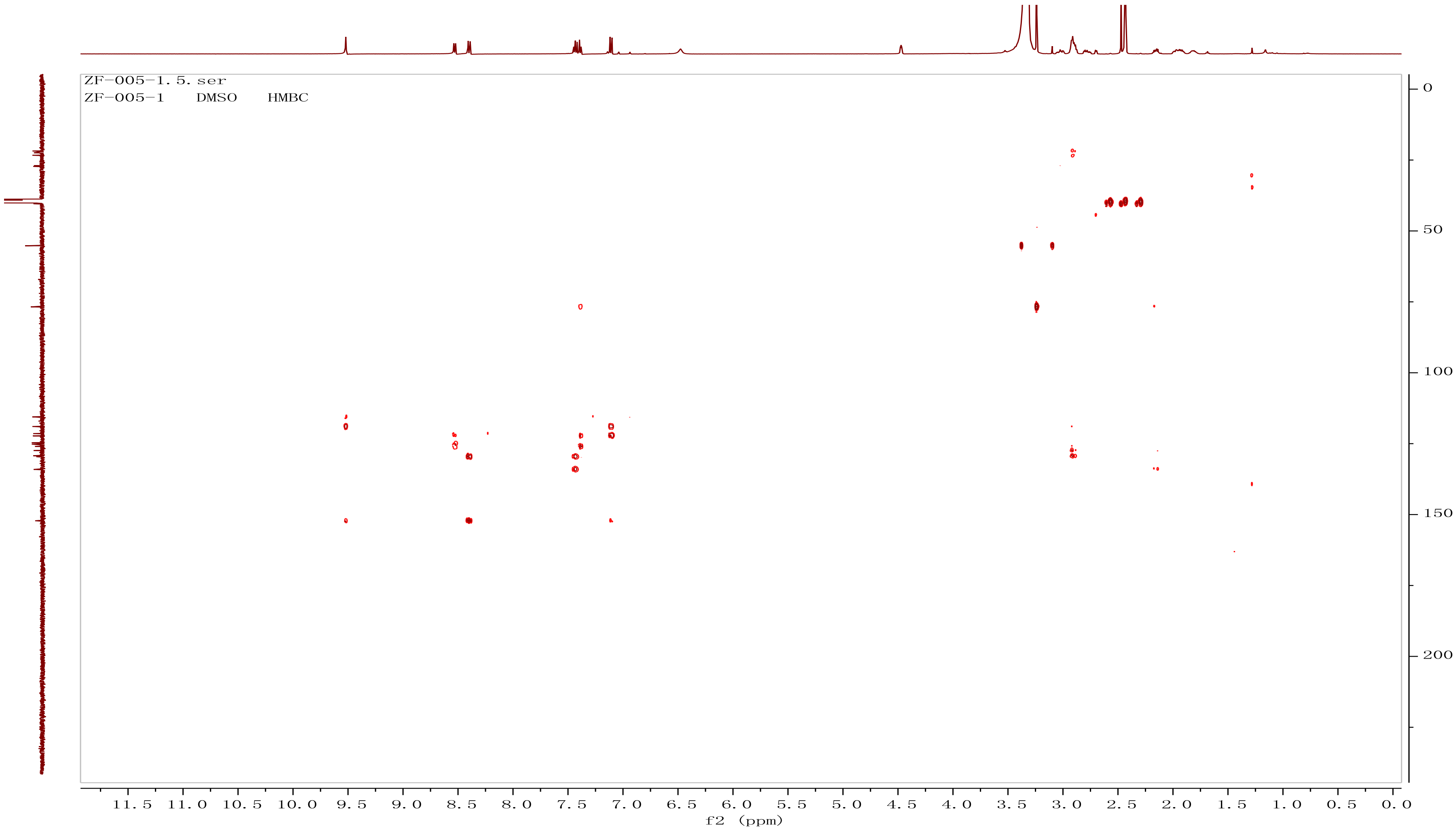

Figure S20. The HMBC Spectrum of Compound **4** in DMSO

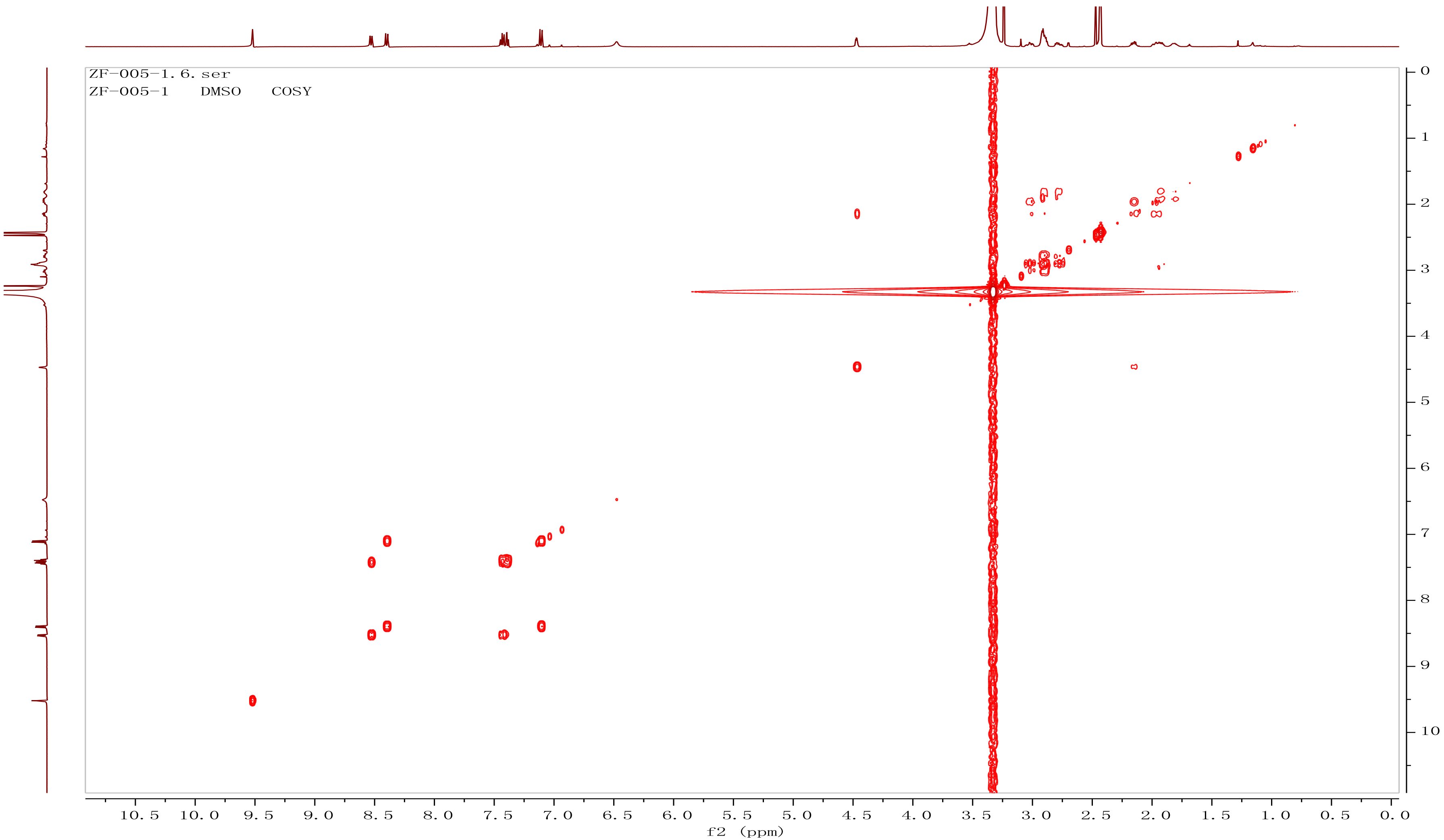

Figure S21. The COSY Spectrum of Compound **4** in DMSO

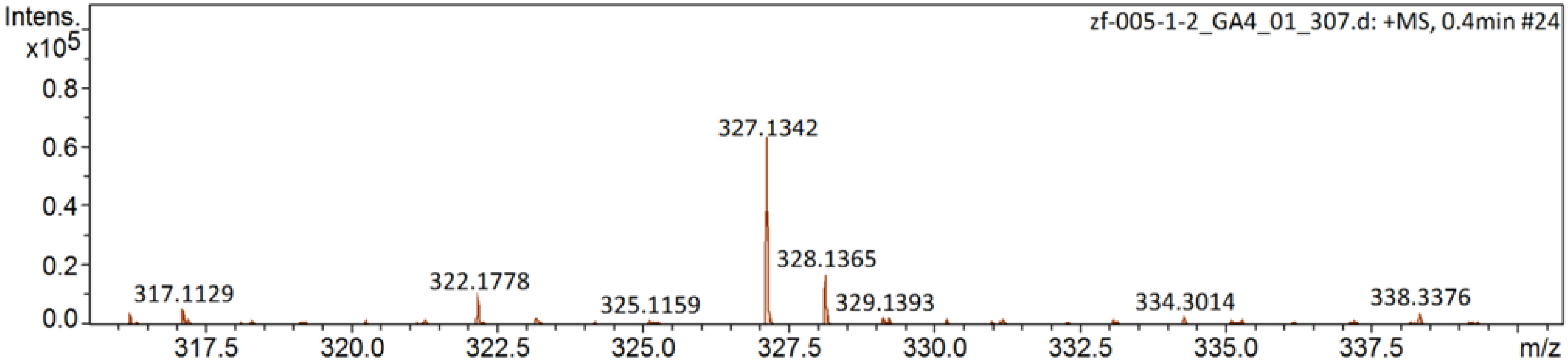

Figure S22. The HRESIMS Spectroscopic Data of Compound **4**

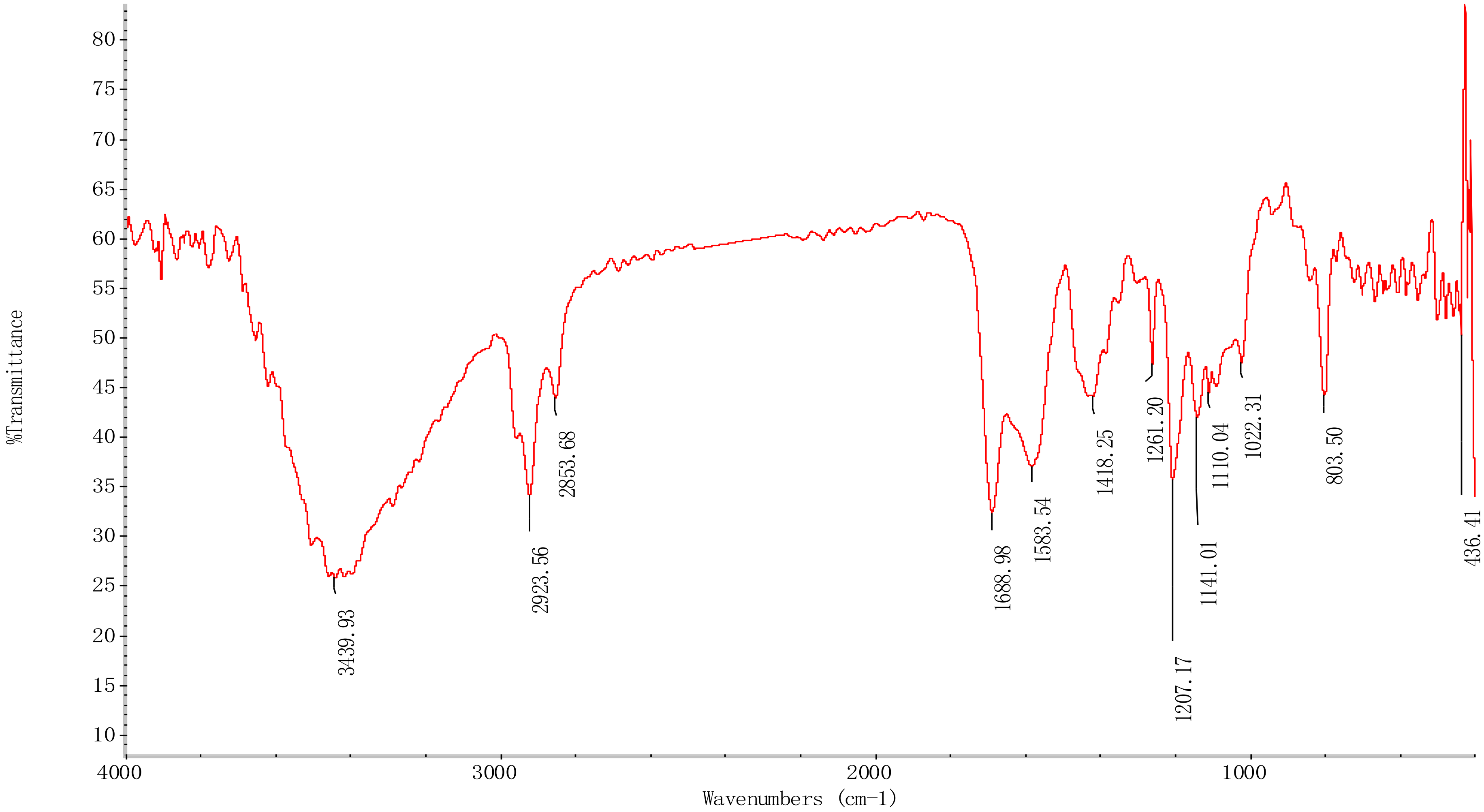

Figure S23. The IR Spectrum of Compound **4**

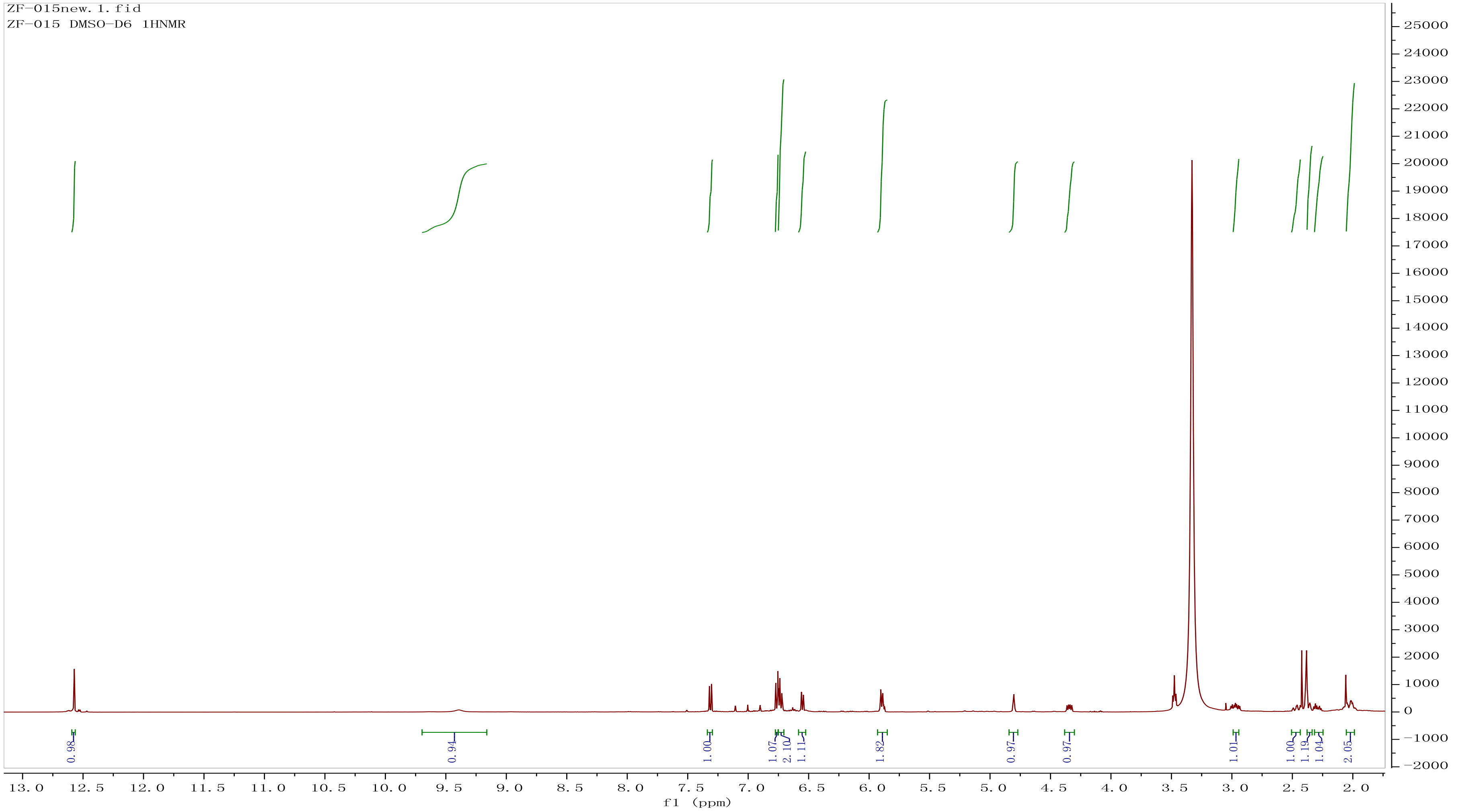

Figure S24. The ^{1}H NMR Spectrum of Compound **5** in DMSO

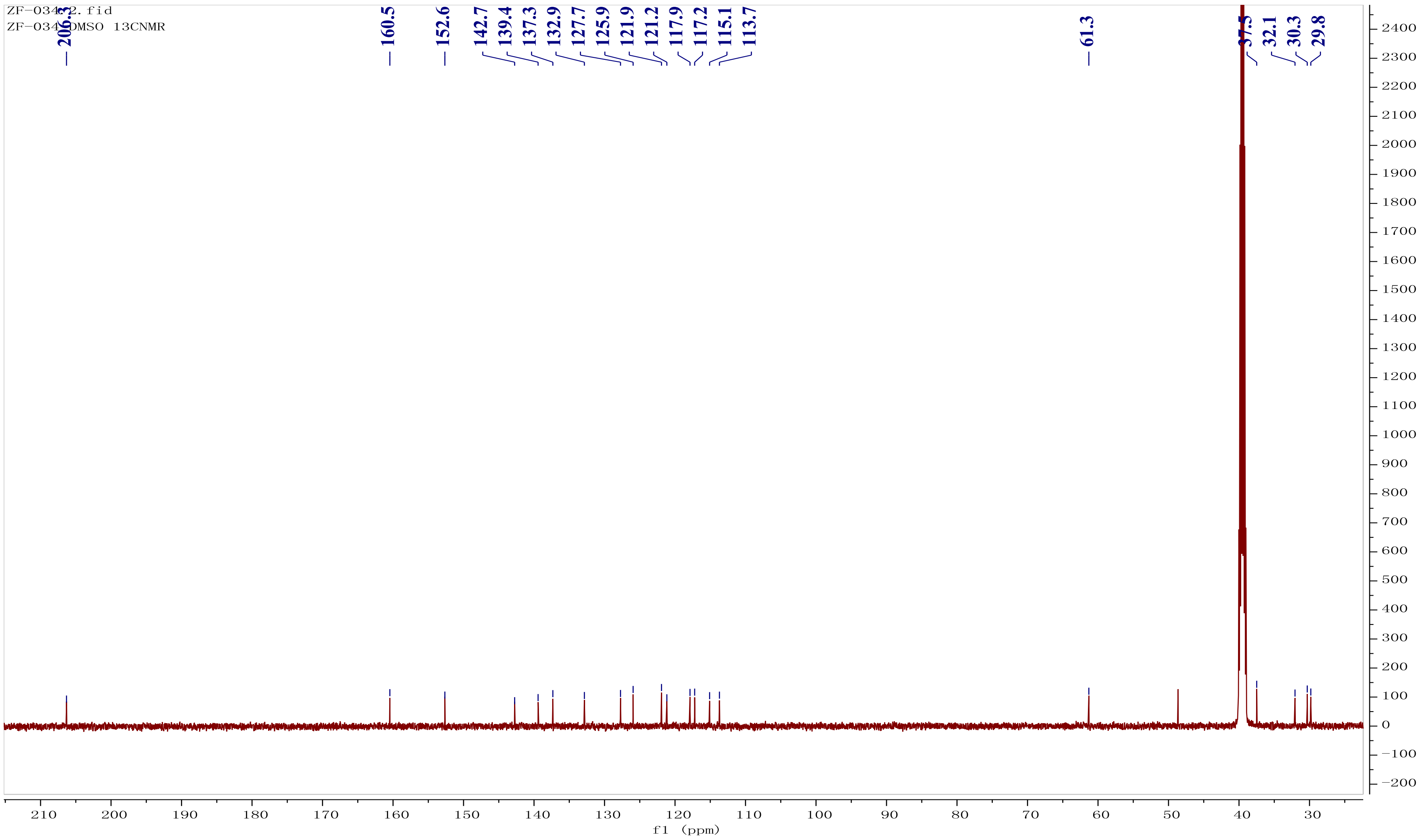

Figure S25. The ^{13}C NMR Spectrum of Compound **5** in DMSO

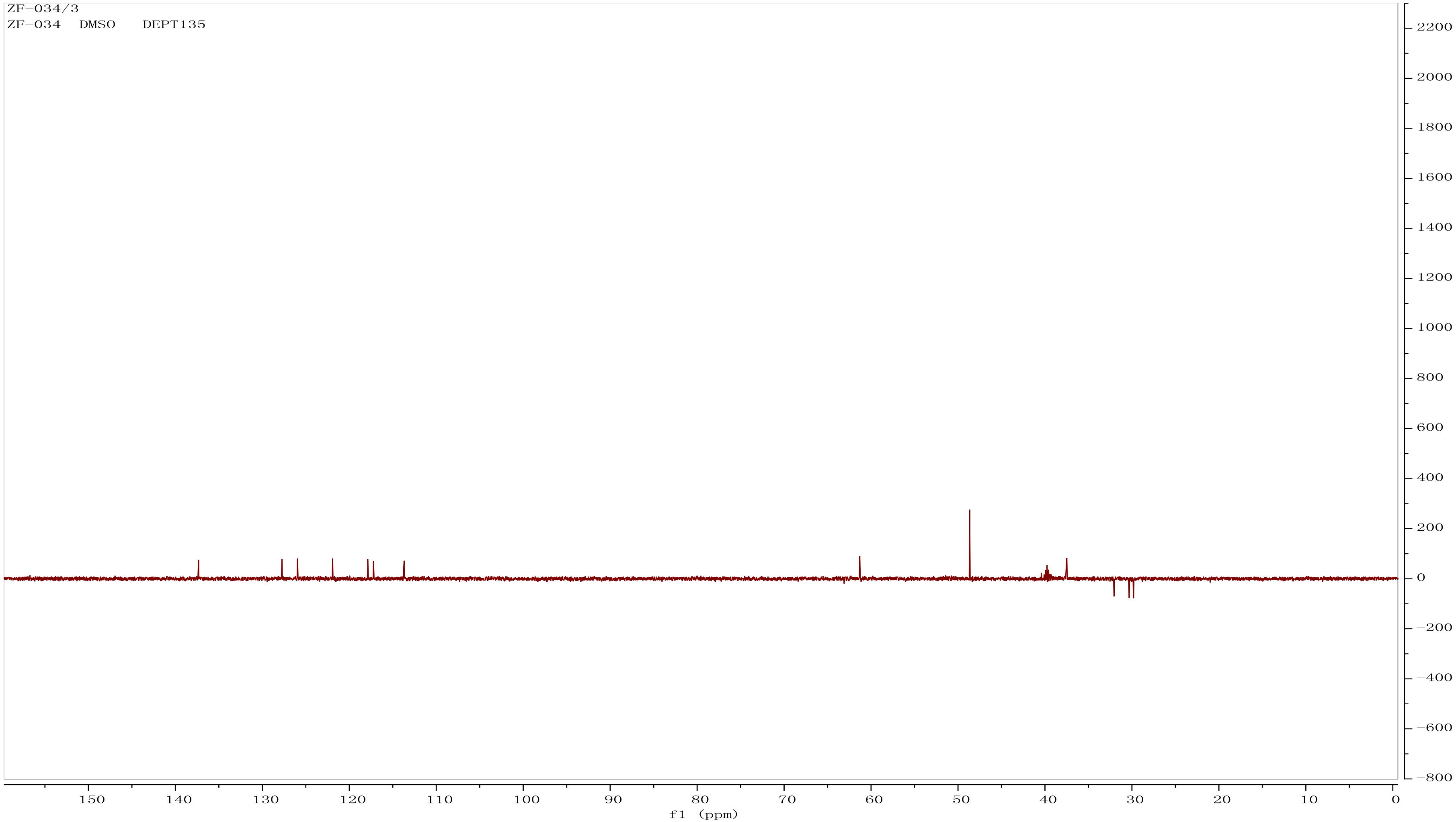

Figure S26. The DEPT Spectrum of Compound **5** in DMSO

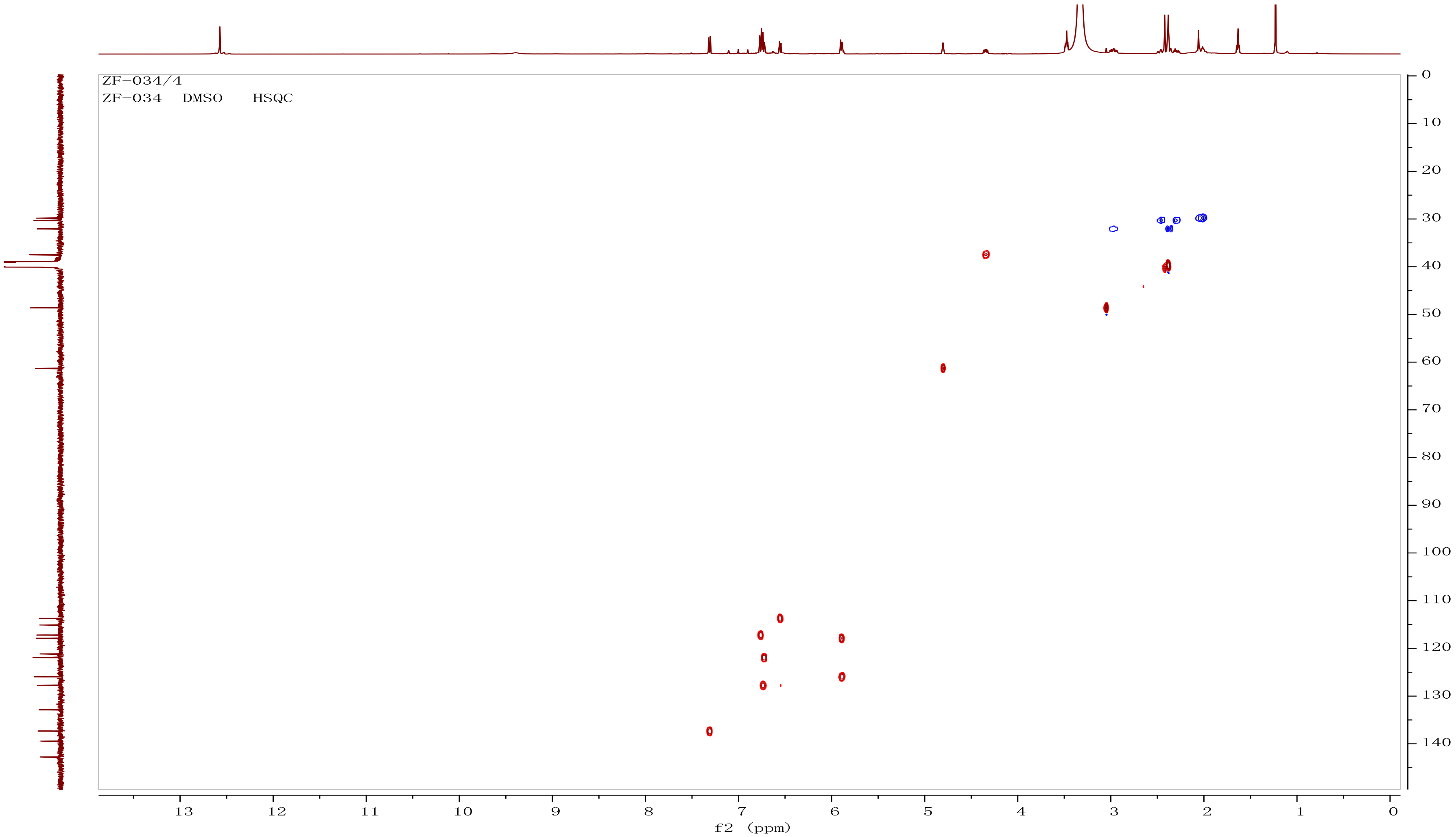

Figure S27. The HMQC Spectrum of Compound **5** in DMSO

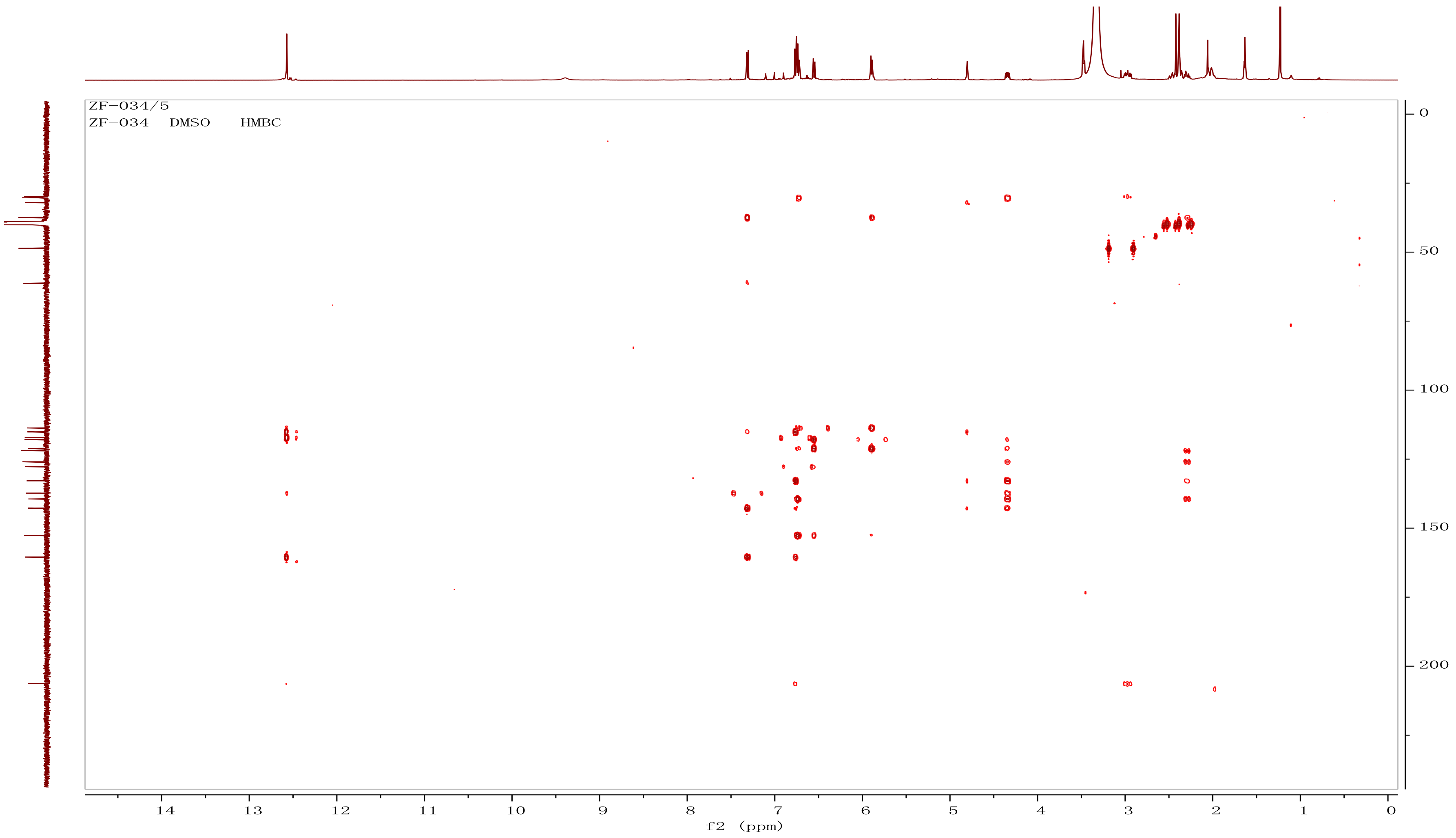

Figure S28. The HMBC Spectrum of Compound **5** in DMSO

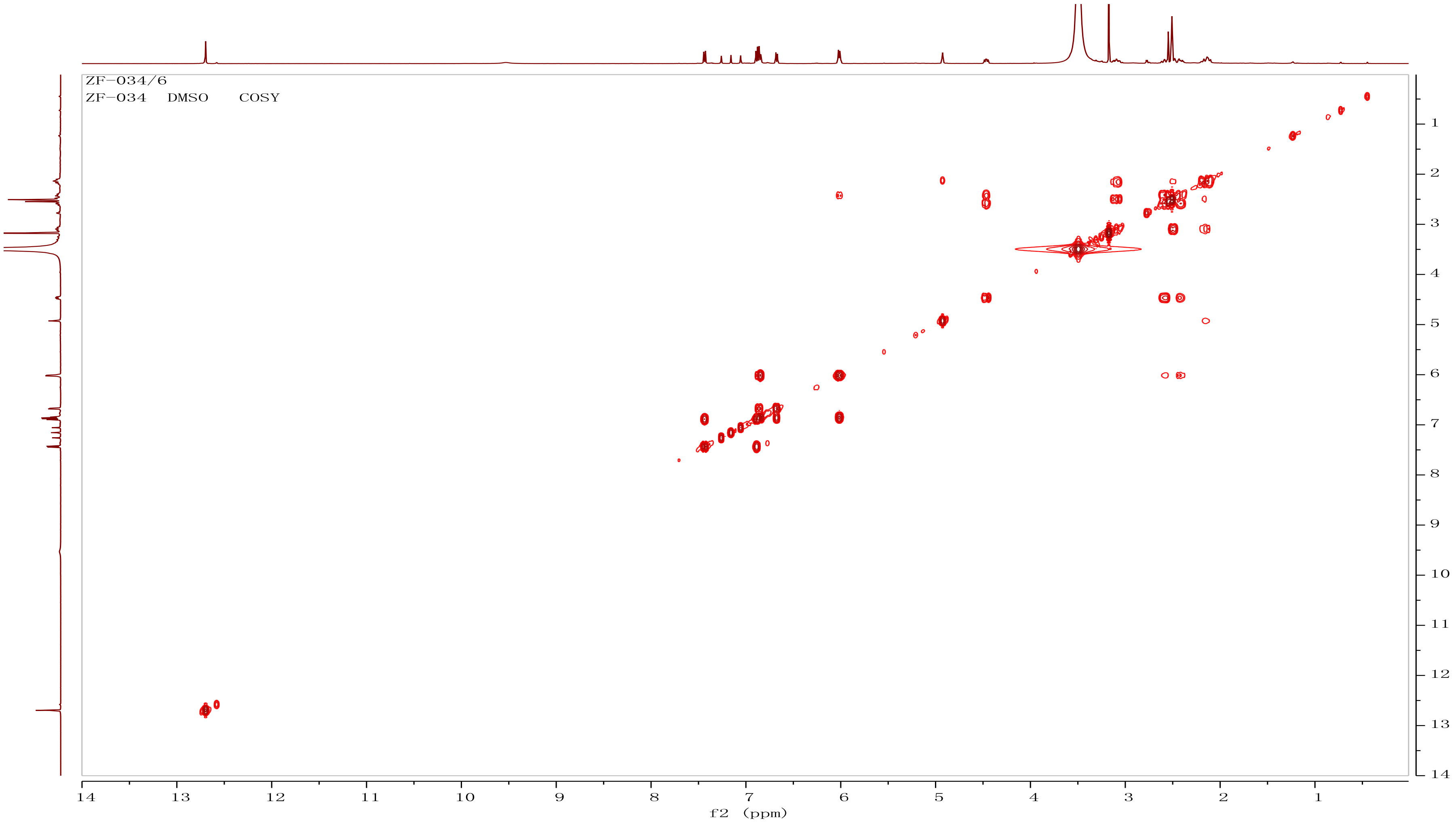

Figure S29. The COSY Spectrum of Compound **5** in DMSO

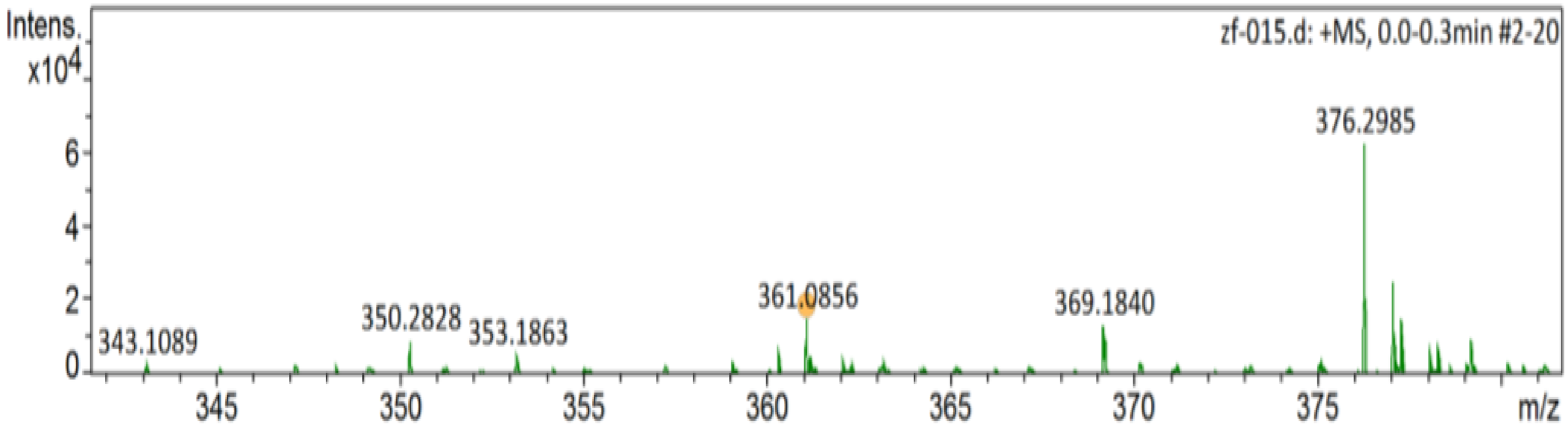

Figure S30. The HRESIMS Spectroscopic Data of Compound **5**

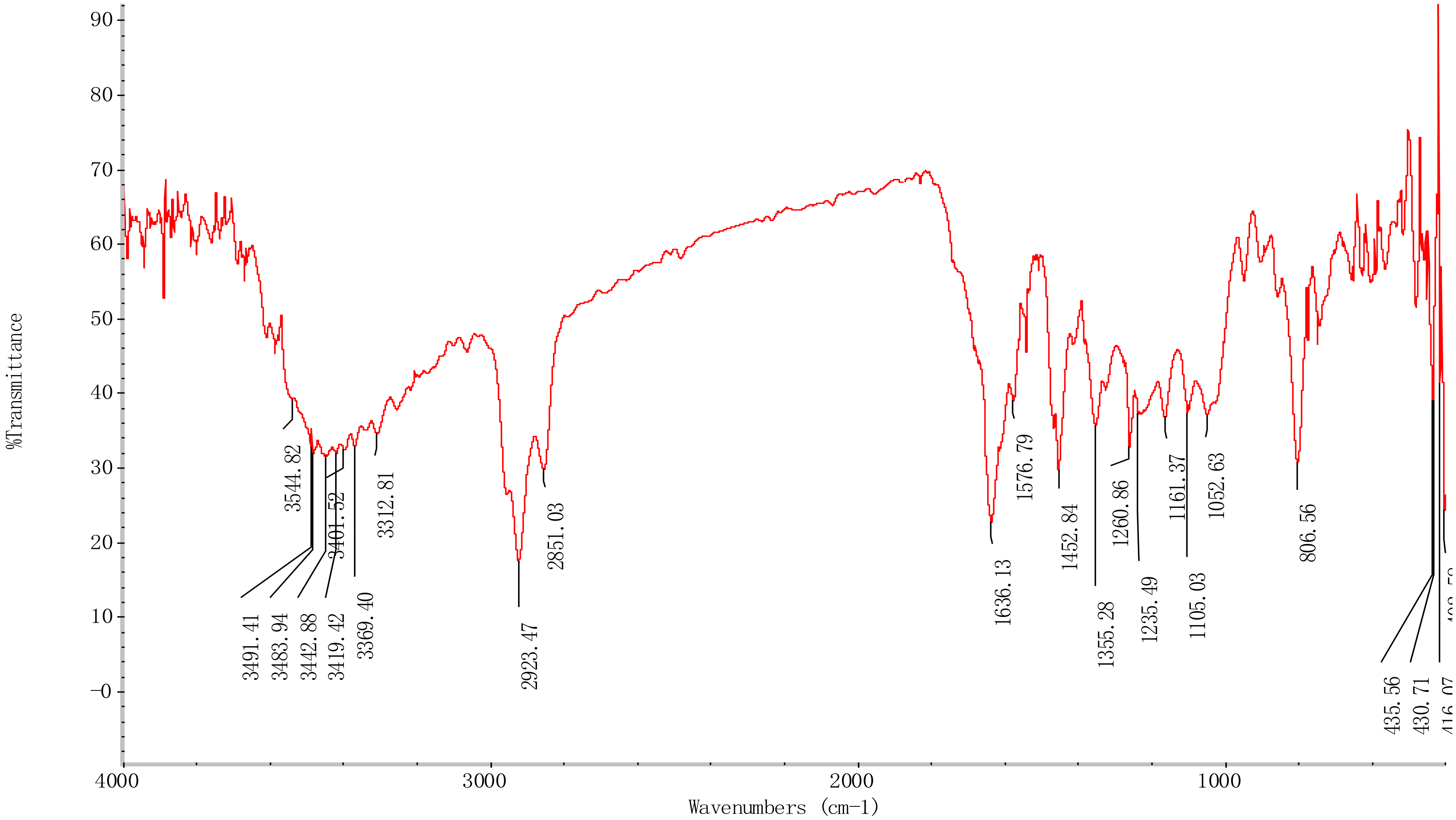

Figure S31. The IR Spectrum of Compound **5**

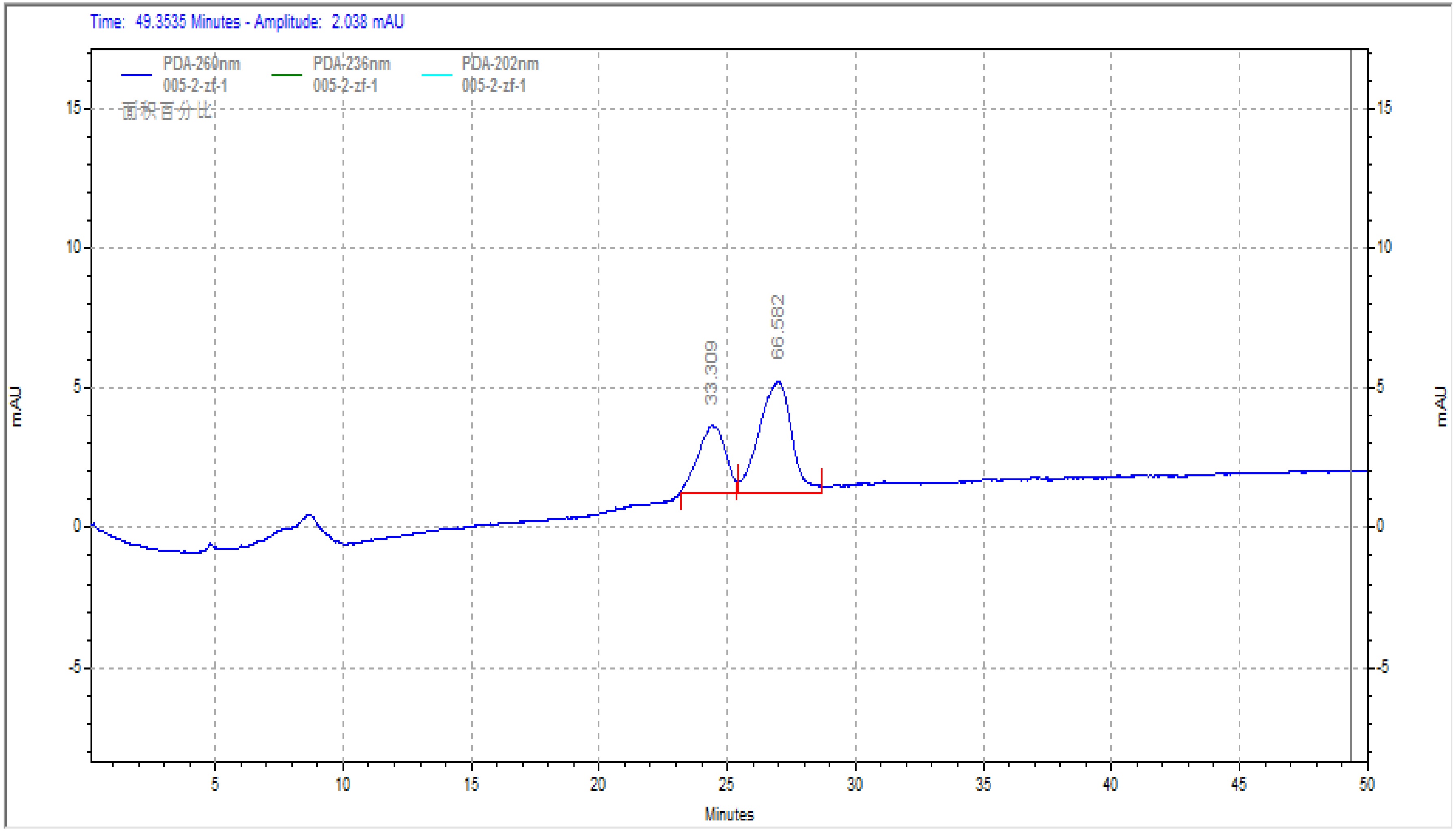

Figure S32. A peak area ratio of compounds **2** and **3** over a chiral column

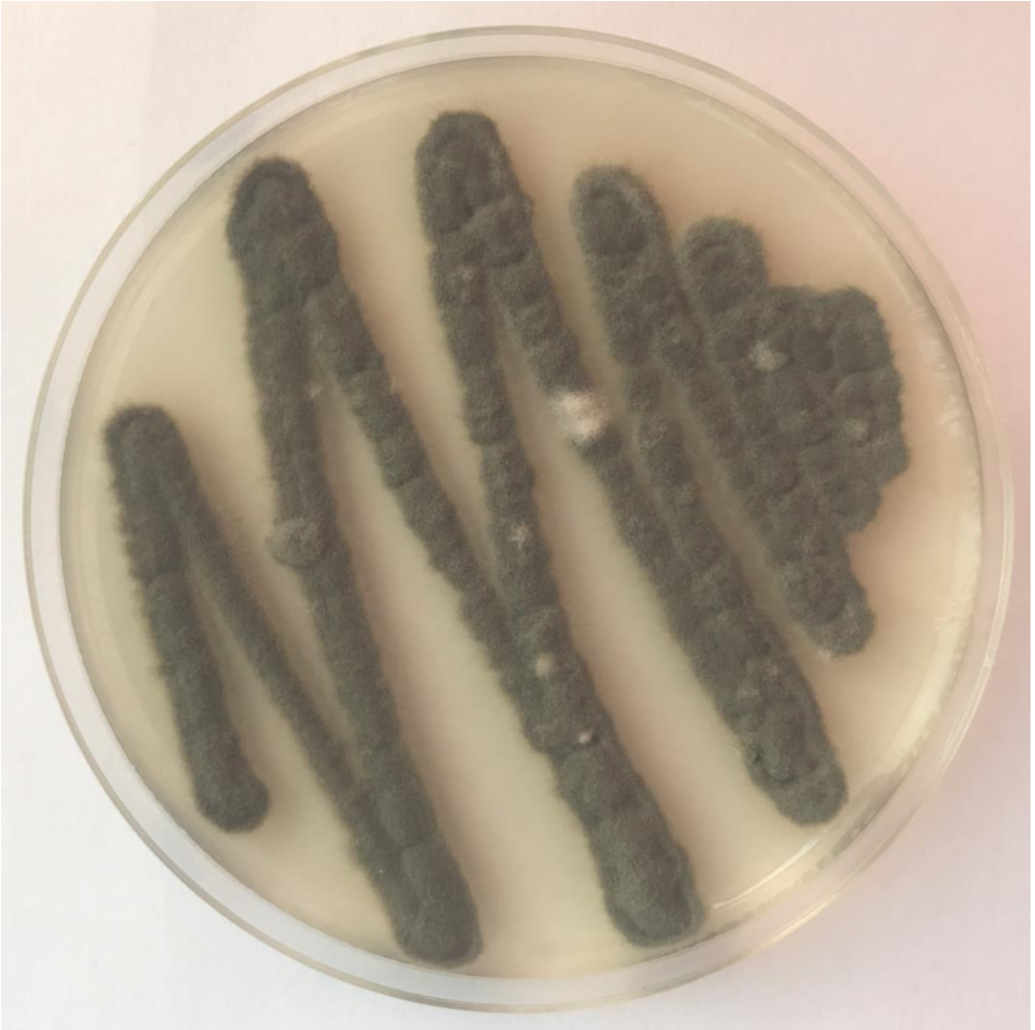

Figure S33. The picture of strain *Cladosporium* sp. KFD33

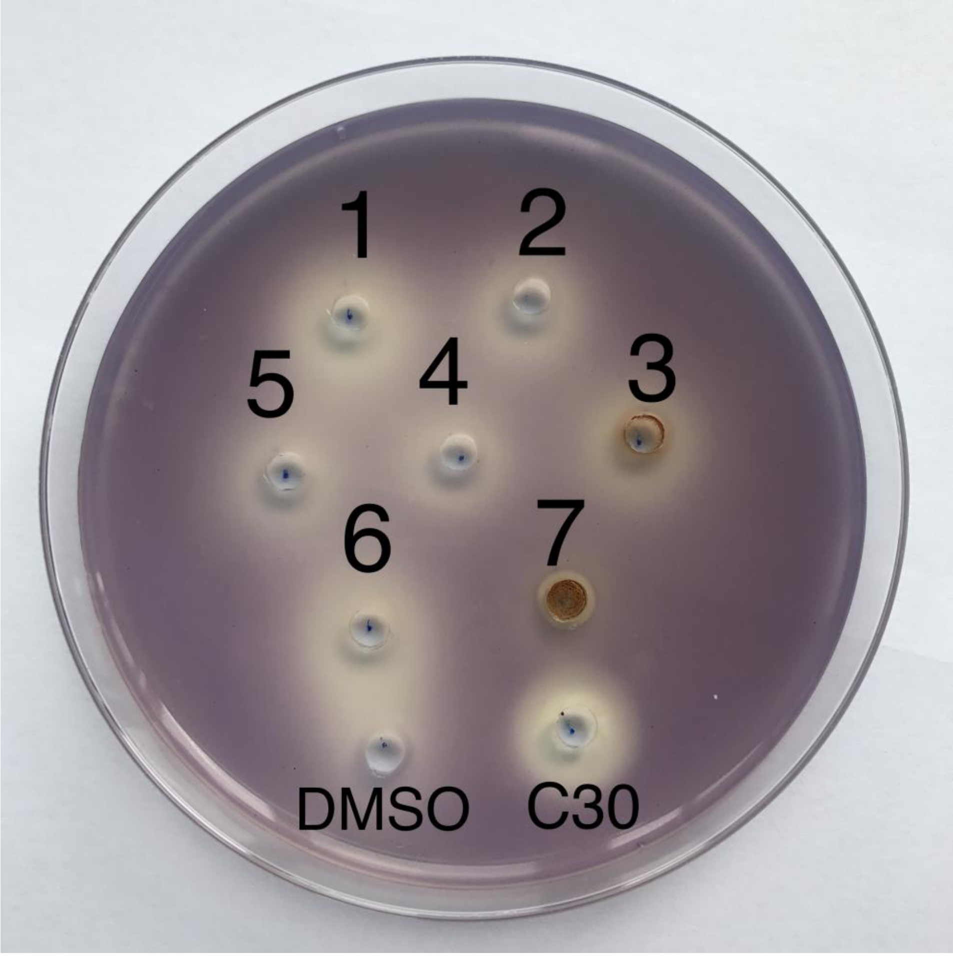

Figure S34. C. *violaceum* CV026 well diffusion assay
(Compounds **1**-**6** with 30, 30, 20, 30, 20 and 30 µg/well)

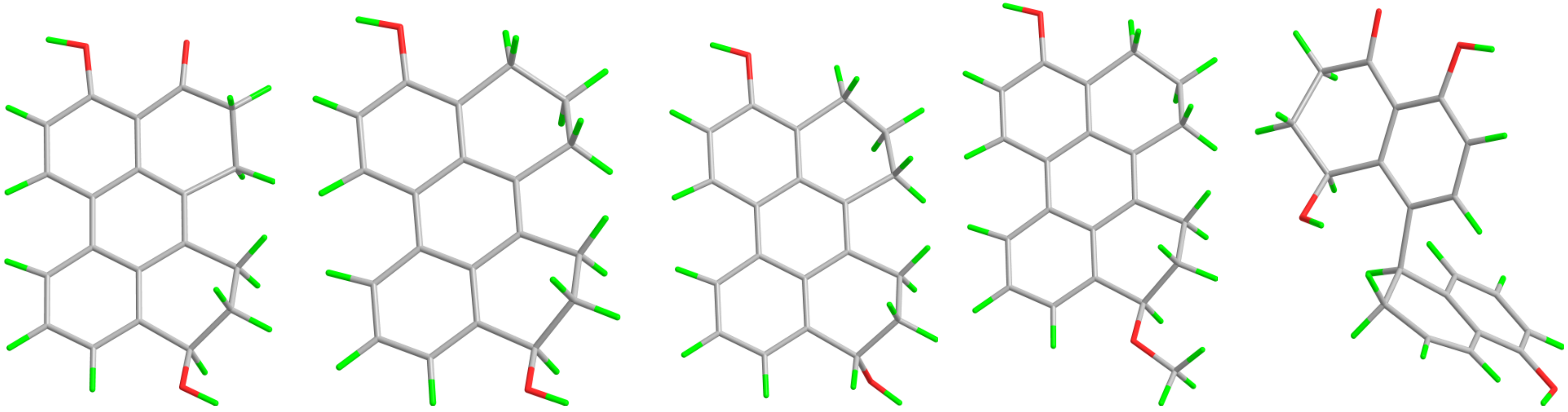

1 2 3 4 5

Figure S35. The energy minimized 3D chemical structures for **1-5**.

18*S* rRNA gene sequences of *Cladosporium* sp. KFD33

GCACTATACGGTGAAACTGCGAATGGCTCATTAAATCAGTTATCGTTTATTTGATAGTACCTTACTACATGGATAACCGTGGTAATTCTAGAG
CTAATACATGCTAAAAACCTCGACTTCGGAAGGGGTGTATTTATTAGATAAAAAACCAATGCCCTTCGGGGCTCCTTGGTGAATCATAATAA
CTTAACGAATCGCATGGCCTTGCGCCGGCGATGGTTCATTCAAATTTCTGCCCTATCAACTTTCGATGGTAGGATAGTGGCCTACCATGGTAT
CAACGGGTAACGGGGAATTAGGGTTCGACTCCGGAGAGGGAGCCTGAGAAACGGCTACCACATCCAAGGAAGGCAGCAGGCGCGCAAATT
ACCCAATCCCGACACGGGGAGGTAGTGACAATAAATACTGATACAGGGCTCTTTTGGGTCTTGTAATTGGAATGAGTACAATTTAAATCCCT
TAACGAGGAACAATTGGAGGGCAAGTCTGGTGCCAGCAGCCGCGGTAATTCCAGCTCCAATAGCGTATATTAAAGTTGTTGCAGTTAAAAA
GCTCGTAGTTGAACCTTGGGCCTGGCTGGCCGGTCCGCCTCACCGCGTGTACTGGTCCGGCCGGGCCTTTCCTTCTGGGGAACCTCATGCCCTT
CACTGGGCGTGTTGGGGAACCAGGACTTTTACTTTGAAAAAATTAGAGTGTTCAAAGCAGGCCTTTGCTCGAATACATTAGCATGGAATAAT
AGAATAGGACGTGTGGTTCTATTTTGTTGGTTTCTAGGACCGCCGTAATGATTAATAGGGATAGTCGGGGGCATCAGTATTCAATCGTCAGAG
GTGAAATTCTTGGATTGATTGAAGACTAACTACTGCGAAAGCATTTGCCAAGGATGTTTTCATTAATCAGTGAACGAAAGTTAGGGGATCGA
AGACGATCAGATACCGTCGTAGTCTTAACCATAAACTATGCCGACTAGGGATCGGACGGTGTTAGTATTTTGACCCGTTCGGCACCTTACGA
GAAATCAAAGTTTTTGGGTTCTGGGGGGAGTATGGTCGCAAGGCTGAAACTTAAAGAAATTGACGGAAGGGCACCACCAGGCGTGGAGCCT
GCGGCTTAATTTGACTCAACACGGGGAAACTCACCAGGTCCAGACACAATAAGGATTGACAGATTGAGAGCTCTTTCTTGATTTTGTGGGTG
GTGGTGCATGGCCGTTCTTAGTTGGTGGAGTGATTTGTCTGCTTAATTGCGATAACGAACGAGACCTTAACCTGCTAAATAGCCAGGCCCGCT
TTGGCGGGTCGCCGGCTTCTTAGAGGGACTATC

The calculations were performed by using the density functional theory (DFT) as carried out in the Gaussian 03.[S1] The preliminary conformational distributions search was performed using Frog2 online version[S2]. Further geometrical optimization were performed at the B3LYP/6-31G(d) level. Solvent effects of methanol solution were evaluated at the same DFT level by using the SCRF/PCM method.[S3] TDDFT[S4] at B3LYP/6-31G(d) was employed to calculate the electronic excitation energies and rotational strengths in methanol.

(S1) Gaussian 03, Revision E.01, M. J. Frisch, G. W. Trucks, H. B. Schlegel, G. E. Scuseria, M. A. Robb, J. R. Cheeseman, J. A. Montgomery, Jr., T. Vreven, K. N. Kudin, J.C. Burant, J. M. Millam, S. S. Iyengar, J. Tomasi, V. Barone, B. Mennucci, M. Cossi, G. Scalmani, N. Rega, G. A. Petersson, H. Nakatsuji, M. Hada, M. Ehara, K. Toyota, R. Fukuda, J. Hasegawa, M. Ishida, T. Nakajima, Y. Honda, O. Kitao, H. Nakai, M. Klene, X. Li, J. E. Knox, H. P. Hratchian, J. B. Cross, V. Bakken, C. Adamo, J. Jaramillo, R. Gomperts, R. E. Stratmann, O. Yazyev, A. J. Austin, R. Cammi, C. Pomelli, J. W. Ochterski, P. Y. Ayala, K. Morokuma, G. A. Voth, P. Salvador, J. J. Dannenberg, V. G. Zakrzewski, S. Dapprich, A. D. Daniels, M. C. Strain, O. Farkas, D. K. Malick, A. D. Rabuck, K. Raghavachari, J. B. Foresman, J. V. Ortiz, Q. Cui, A. G. Baboul, S. Clifford, J. Cioslowski, B. B. Stefanov, G. Liu, A. Liashenko, P. Piskorz, I. Komaromi, R. L. Martin, D.J. Fox, T. Keith, M. A. Al-Laham, C. Y. Peng, A. Nanayakkara, M. Challacombe, P. M. W. Gill, B. Johnson, W. Chen, M. W. Wong, C. Gonzalez, and J. A. Pople, Gaussian, Inc., Wallingford CT, 2004.

(S2) Miteva, M. A.; Guyon, F.; and Tuffery, P.; *Nucleic Acids Res.*, **2010**, *38*, W622–W627.

(S3) Sai, C.; Li, D.; Xue, C.; Wang, K.; Hu, P.; Pei, Y.; Bai, J.; Jing Y.; Li, Z.; Hua H. *Org. Lett.* **2015**, *17*, 4102-5.

(S4) (a) Miertus, S.; Tomasi, *J. Chem. Phys.* **1982**, *65*, 239–245. (b) Tomasi, J.; Persico, M. *Chem.Rev.* **1994**, *94*, 2027–2094. (c) Cammi, R.; Tomasi, J. J. *Comp.Chem.* **1995**, *16*, 1449–1458.
